# Piezoelectric Materials for Controlling Electro-Chemical Processes

**DOI:** 10.1007/s40820-020-00489-z

**Published:** 2020-07-14

**Authors:** Weiqi Qian, Weiyou Yang, Yan Zhang, Chris R. Bowen, Ya Yang

**Affiliations:** 1 CAS Center for Excellence in Nanoscience, Beijing Key Laboratory of Micro-nano Energy and Sensor, Beijing Institute of Nanoenergy and Nanosystems, Chinese Academy of Sciences, Beijing, 100083 People’s Republic of China; 2 Institute of Materials, Ningbo University of Technology, Ningbo, 315211 People’s Republic of China; 3 Department of Mechanical Engineering, University of Bath, Bath, BA2 7AK UK; 4 School of Nanoscience and Technology, University of Chinese Academy of Sciences, Beijing, 100049 People’s Republic of China; 5 Center on Nanoenergy Research, School of Physical Science and Technology, Guangxi University, Nanning, 530004 People’s Republic of China

**Keywords:** Piezoelectric materials, Piezoelectric effect, Electro-chemistry, Piezo-electro-chemistry

## Abstract

Piezoelectric materials have been analyzed for over 100 years, due to their ability to convert mechanical vibrations into electric charge or electric fields into a mechanical strain for sensor, energy harvesting, and actuator applications. A more recent development is the coupling of piezoelectricity and electro-chemistry, termed piezo-electro-chemistry, whereby the piezoelectrically induced electric charge or voltage under a mechanical stress can influence electro-chemical reactions. There is growing interest in such coupled systems, with a corresponding growth in the number of associated publications and patents. This review focuses on recent development of the piezo-electro-chemical coupling multiple systems based on various piezoelectric materials. It provides an overview of the basic characteristics of piezoelectric materials and comparison of operating conditions and their overall electro-chemical performance. The reported piezo-electro-chemical mechanisms are examined in detail. Comparisons are made between the ranges of material morphologies employed, and typical operating conditions are discussed. In addition, potential future directions and applications for the development of piezo-electro-chemical hybrid systems are described. This review provides a comprehensive overview of recent studies on how piezoelectric materials and devices have been applied to control electro-chemical processes, with an aim to inspire and direct future efforts in this emerging research field.

## Highlights

- This review focuses on recent development of the piezo-electro-chemical coupling multiple systems based on various piezoelectric materials.
- Comparison of operating conditions and their electro-chemical performance is provided.
- Challenges, potential future directions, and applications for the development of piezo-electro-chemical hybrid systems are described.

## Graphical abstract

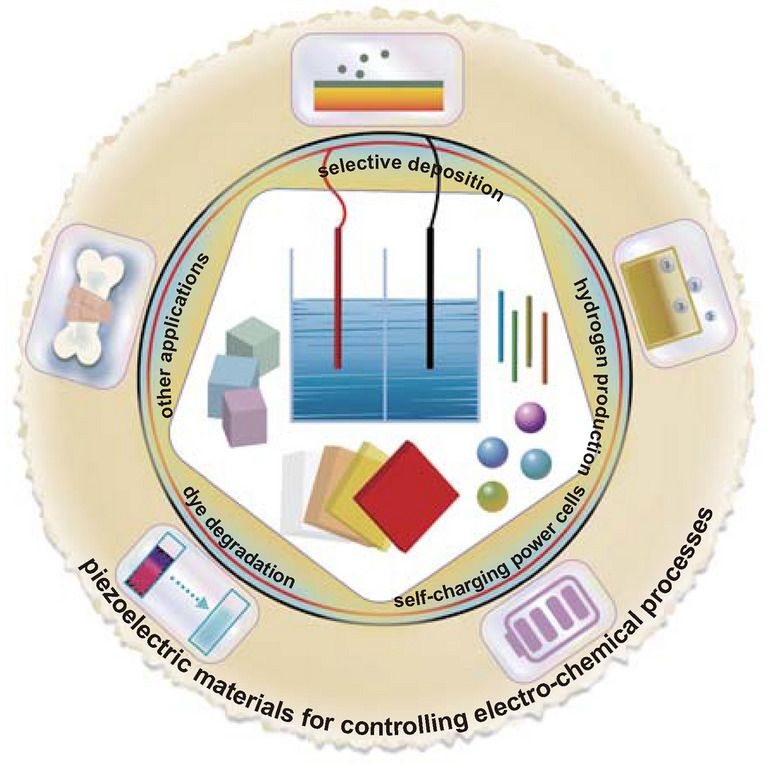

## Introduction

Piezoelectricity was first discovered by P. Cure and J. Curie in 1880 based on their observations of the production of an electrical charge when specific materials were subjected to a mechanical force [1]. The term ‘piezoelectricity’ originates from ‘*piezo*’ and ‘*electricity*,’ where ‘*piezo*’ represents the application of a pressure and ‘*electricity*’ corresponds to moving electrons [2]. Materials that exhibit piezoelectricity are termed *piezoelectric* materials, which generate an electric charge in response to applied stress (the *direct* piezoelectric effect), and develop a mechanical strain when subjected to an applied electric field (the *converse* piezoelectric effect) [3–7].

The origin of piezoelectricity is related to a non-centrosymmetric distribution of positive and negative electric charges in the unit cell of a material [8, 9]. When a piezoelectric material is subjected to an applied stress or mechanical vibration, the induced displacement of ions results in a net electric charge due to a change in the dipole moment of the unit cell, which builds a piezoelectric potential across the material [10, 11]. Generally, among the 21 crystal point groups of non-centrosymmetric crystals, there are 20 point groups of crystals possessing piezoelectricity, where 10 point groups belong to nonpolar crystals which show piezoelectricity and the other 10 point groups of polar crystals exhibit piezoelectricity and ferroelectricity [8, 9]. Piezoelectric materials belonging to nonpolar crystals which are non-ferroelectric can have no electric net dipole in the zero-stress state and only generate an electric dipole under stress due to the separation of electric charge centers and a resulting induced piezoelectric potential; a good example of such a material is quartz [12–18]. There are also piezoelectric materials belonging to polar crystals that exhibit a spontaneous polarization in the zero-stress state or no electric field state since there is a separation between positive and negative charges [19, 20]. A good example of such a material is zinc oxide. A subclass of piezoelectric materials are *ferroelectric* materials belonging to polar crystals, whose spontaneous polarization can be changed permanently and switched when exposed to an external strong electric field, for example, in barium titanate [21, 22]. Since the polarization of a ferroelectric changes with stress, all ferroelectric materials exhibit piezoelectricity by default [11, 23, 24].

Irrespective of the mechanism by which polarization is induced, whether spontaneously or mechanically, the induced electric field across material affects its electrical properties dramatically on the interior and the exterior regions of the material, where the built-in electric field can disrupt electronic energy states, and electric charges are rearranged [25, 26]. If the outside surface of the material is in contact with a medium, the rearrangement of electric charges can alter the electric conductivity, which is highly dependent on the density of electric charge as well as the continuity of the occupiable electronic energy states between the material and the medium [27]. We will see in the review that this process can have a particularly strong influence on electro-chemistry.

Typically, an electro-chemical reaction is driven by an external power source [28–30], and the coupling of power generation with electro-chemical process remains a vibrant topic. Large-scale renewable and clean power generation approaches are being considered that store solar and wind energy and subsequently convert it into electrical power for driving electro-chemistry [31–34]. However, smaller-scale and more local energy, such as mechanical energy in the range of microwatt to milliwatt, can be harvested and utilized by systems based on piezoelectric materials [19, 35]. In recent decades, piezoelectrically induced electric fields have been used to control catalytic rates in chemical solutions [36–38], the corrosion rate of metals in etchant solutions [39–43], self-charging systems [44–50], and a variety of other electro-chemical processes [51–53]. The coupling of piezoelectricity and electro-chemistry is termed *piezo*-*electro*-*chemistry*, where a piezoelectrically induced electric charge or potential difference generated by a mechanical stress can influence electro-chemical reaction systems [54–56].

**Fig. 1:**
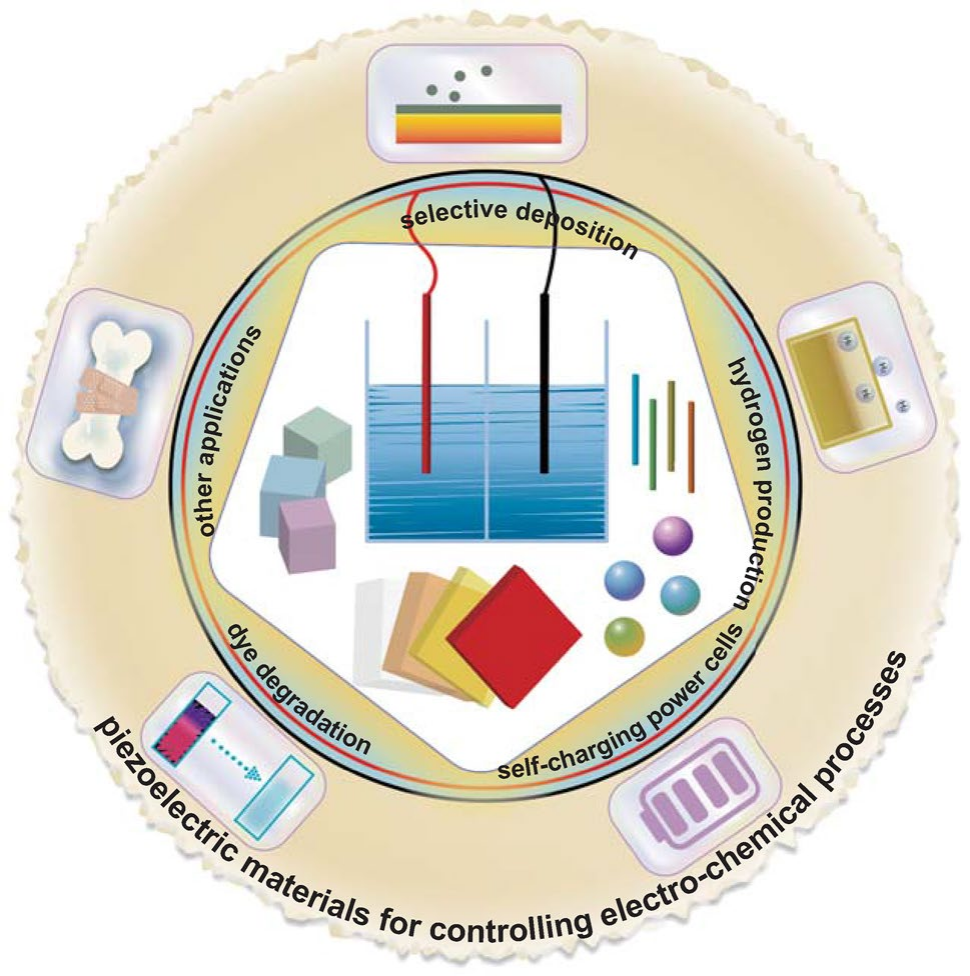
Piezo-electro-chemical reaction systems to be covered in the review with various materials and practical applications

There are a variety of excellent reviews on electro-chemistry [19, 57–64], but those that specifically focus on piezoelectrically influenced electro-chemical reactions have received limited attention to date. This review places a focus on the range of piezoelectric materials used for controlling electro-chemical processes. It will provide an overview of the basic characteristics of piezoelectric materials and comparison of the operating conditions and electro-chemical performance. The reported piezo-electro-chemical mechanisms will then be examined in detail. Within this review, we have collected virtually all published research work to date on the use of piezoelectric materials for controlling electro-chemistry; this body of work is summarized in Table 1 which contains detailed information regarding the specific piezoelectric materials, along with the electro-chemical processes and performance. In addition, the piezo-electro-chemical reaction systems to be covered within this review include materials that are in bulk [65, 66], fiber [67–69], sheet [70, 71], flower [37, 72, 73], particle [74, 75], and irregular [32, 76] form. The piezoelectric materials include ferroelectric perovskites [77, 78], wurtzite zinc oxide [79, 80], two-dimensional layered transition metal dichalcogenide-based materials [81, 82], organic piezoelectric materials [44, 83, 84], and biological materials [85] that are used for a variety of applications such as selective deposition [38, 77, 86], hydrogen production [32, 65, 69], dye degradation [73, 76, 87–89], self-charging power cells [44, 45, 49, 83], and others [47, 90]. The above-mentioned piezo-electro-chemical reactions are shown schematically in Fig. 1, and the intention of this review is to overview recent studies on piezoelectric materials and devices that have been applied to control electro-chemical processes and inspire increasing efforts in this new and emerging research field.

**Table 1:** Overview of piezoelectric materials and their individual characteristics for piezo-electro-chemical processes

Materials	Piezoelectric material characteristics						Piezo-electro-chemical processes			Refs.
	Synthesis method	Polarization	Shape	Size	Piezoelectric constant	Piezoelectrically induced potential	Application(s)	Conditions	Performance	
PMN-PT	Commercially obtained	Poled along the <001> direction	Slab	24 × 4 × 0.25 mm^3^	2200–2700 pC/N	20 V	Hydrogen production	Vibrator (10 and 20 Hz) and linear actuator	~ 0.01–0.08 ppb/oscillation	[65]
PZT	Sol–gel process	Polarized under 14 V by AFM	Grains	70–100 nm	–	–	Selective deposition	Hg lamp (400 W)	Metal ions can be reduced to metal by photoexcited e^−^ at the surface of *c*^+^ domains	[179]
PZT	Sol–gel process	Polarized under 12 V by AFM	Films	Thickness: 70 nm; area: 2 cm^2^	–	–	Selective deposition	Hg lamp and Fe-doped Hg lamp (400 W)	Deposition of silver on *c*^+^ domains	[77]
PZT	Commercially obtained	Two PZT ceramics polarized opposite	Wafer	30 × 15 × 0.3 mm^3^	~500 pC/N	~12 V	Hydrogen production	A cyclic force of ~ 0.07 N; a resonance frequency of ~ 46.2 Hz	~10^−8^ mol/min	[66]
PZT	Hydrothermal reaction	No poling	Fibers	Diameter: ~ 500 nm; length: several micrometers	–	–	Dye degradation	Ultrasonic mechanical vibration (5.05 × 10 kPa; 40 kHz)	Degradation ratio of 80% for acid orange 7 solutions (30 μmol/L)	[67]
TiO_2_/BFO	Ball-milling method	Polarized by AFM	Substrates	–	–	–	Selective deposition	UV light	Reduction of aqueous silver cations from solution	[38]
BFO	Hydrothermal reaction	No poling	Square micro-sheets	~ 1 μm	~ 70 pm/V	–	Dye degradation	Under an ultrasonic source (5.05 × 10 kPa; ~ 40 kHz)	Degradation ratio of ~ 95% for rhodamine B solutions (~ 10 mg/L)	[70]
BFO	Hydrothermal reaction	No poling	Square micro-sheets	~ 380 nm	~ 100 pm/V	~ 0.88 V	Hydrogen production	Under a mechanical vibration excitation for 1 h (100 W 1.01 × 10^5^ kPa; ~ 45 kHz)	Hydrogen production rate of ~ 124.1 μmol/g	[71]
							Dye degradation		Degradation ratio of ~ 94.1% for rhodamine B solutions within 50 min	
BFO	Hydrothermal reaction	No poling	Nanosheets	Area: 2–3 μm; thickness: ~ 150 nm	~ 100 pm/V	–	Dye degradation	UV–visible light and ultrasonic mechanical vibrations	Degradation ratio of ~ 71% for rhodamine B solutions within 1 h	[78]
			Nanowires	Length: 30 μm; diameter: 200–700 nm					Degradation ratio of ~ 97% for rhodamine B solutions within 1 h	
BFO-PDMS	Hydrothermal reaction	No poling	Nanoflowers	~ 30 μm	70 pm/V	–	Dye degradation	Ultrasonic mechanical vibrations (40 kHz, 400 W)	Degradation ratio of ~ 98% for rhodamine B solutions (40 mL, 5 mg/L)	[37]
BTO	Molten salt flux method	No poling	Particles	1–5 μm	–	–	Selective deposition	Hg lamp (300 W)	Apparent dependence on the surface orientation ((100) > (111) > (110))	[74]
BTO	Hydrothermal reaction	No poling	Microdendrites	~ 10-μm-long rods with a-few-micrometer-long secondary branches	–	–	Hydrogen production	Ultrasonic mechanical vibrations	1.25 × 10^−2^ ppm/s	[32]
BTO–TiO_2_	High-temperature calcination	Polarized by AFM	Substrates	~ 50 μm	–	–	Selective deposition	UV light	Patterning of products on the film surface, reproducing patterns of products on the bare substrate	[74]
BTO	Hydrothermal reaction	No poling	Microdendrites	~ 10-μm-long rods with a-few-micrometer-long secondary branches	–	–	Dye degradation	Ultrasonic mechanical vibrations (40 kHz)	Degradation ratio of ~ 80% for acid orange 7 solutions (5.7 × 10^−5^ M) within 90 min	[32]
Ag_2_O-BTO	Chemical precipitation	No poling	Nanocubes	~ 50 nm	–	–	Dye degradation	Ultrasonic mechanical vibrations (40 kHz, 50 W) and UV light irradiation	Total degradation for rhodamine B solutions within 1.5 h	[36]
Si/CNT/BTO	High-energy ball-milling process	Poling BTO to create a piezoelectric potential	Nanocomposite particles	< 100 nm	350 pC/N	–	Li-ion batteries	Deformation of Si nanoparticles during lithiation (1.7 GPa)	Coulombic efficiency converged to 98% by the fifth cycle and increased to 99.8% at around the hundredth cycle	[49]
BTO	Hydrothermal reaction	No poling	Microcrystals with a coral-type surface texture	Coral branches with a diameter of 200−400 nm	–	–	Dye degradation and dechlorination	Ultrasonic mechanical vibrations (40 kHz) and ferrous ions added	Degradation ratio of 93.4% for acid orange 7 solutions (5.7 × 10^−2^ mmol/L, 5 mL, pH 3.0)	[76]
BTO	Hydrothermal reaction	No poling	Particles	32.5 nm	–	–	Dechlorination	Ultrasonic mechanical vibrations (40 kHz, 110 W)	Dechlorination ratio of 35.2% and degradation ratio of 71.1% for 4-chlorophenol solutions (25 mg/L)	[75]
BTO	Hydrothermal reaction	No poling	Nanowires	Diameter: 100 nm; length: a few micrometers	–	–	Dye degradation	Ultrasonic mechanical vibrations (40 kHz, 80 W)	Effective enhancement degradation in BTO nanowires for methyl orange solutions within 160 min (100 mL, 5 mg/L)	[101]
			Nanoparticles	200 nm						
BTO–PDMS	Electrospinning	No poling	Particles	< 1 μm	180 pm/V	–	Dye degradation	Ultrasonic mechanical vibrations (40 kHz, 400 W)	Degradation ratio of ~ 94% for rhodamine B solutions (40 mL, 5 mg/L)	[37]
ZTO	Hydrothermal reaction	No poling	Nanowires	Length: 500 nm	–	–	Dye degradation	UV irradiation (15 W) and pressured by an array of multiple stress probes	~ 27% degradation improvement in piezo-photocatalysis for methylene blue solutions (4 ppm)	[68]
ZTO	Hydrothermal reaction	No poling	Nanowire arrays	Dozens of micron	–	–	Dye degradation	UV irradiation (320–340 nm, 30 W), ultrasonic mechanical vibrations, and a piece of glass	Piezophotodegradation rate of ~ 1.5 × 10^−2^ min^−1^ for methylene blue solutions (10 mL, 4 ppm)	[89]
ZnO	Hydrothermal reaction	No poling	Fibers	Diameter: ~ 0.4 μm; length: 4–10 μm	–	–	Hydrogen production	Ultrasonic mechanical vibrations	3.4 × 10^−3^ ppm/s	[32]
Ag/Ag_2_S–ZnO/ZnS	Modified polyol process	No poling	Nanorods	Length: > 100 nm	–	1 V	Hydrogen production	Xenon arc lamp (300 W, 100 mW/cm^2^) and ultrasonic mechanical vibrations	1250 μmol h^−1^ g^−1^	[69]
							Dye degradation		Highest rate constant of 0.0224 min^−1^ for methyl orange solutions	
ZnO	Hydrothermal reaction	No poling	Nanowire arrays	Length: 1600 nm; diameter: 50 nm	–	~ 0.4 V	Piezoelectric nanogenerator	External 500 Pa pressure	Close circuit current peak reached ~ 2 nA	[47]
							Supercapacitor	External 3 mV power supply for 0.1 s	Close circuit current peak reached ~ 2 nA	
CuS/ZnO	Hydrothermal reaction	No poling	Nanowires	Diameter: ~ 100 nm; length: ~ 4 μm	–	–	Dye degradation	Xenon lamp (500 W, 200−1100 nm) and ultrasonic probe (200 W)	Complete degradation for methylene blue solutions (50 mL, 5 mg/L) within 20 min	[80]
ZnO/C	Hydrothermal reaction	No poling	Nanowires	Diameter: 500 nm; length: 6 μm	–	20 mV	Dye degradation	UV irradiation (50 W, 313 nm) and periodically applied force (1 Hz, 1 cm)	Degradation ratio of ~ 96% for methylene blue solutions (100 mL, 5 mg/L) within 120 min	[88]
Ag_2_O/ZnO	Thermal evaporation	No poling	Tetrapod structure	Diameter: ~ 200 nm; leg length: ~ 4 μm	–	–	Dye degradation	UV irradiation (50 W) and ultrasonic probe (200 W)	Degradation ratio of 99% for methylene blue solutions (100 mL, 5 mg/L) within 2 min	[79]
ZnO@TiO_2_	Hydrothermal reaction	No poling	Nanofibers	Diameter: ~ 20 nm; length: ~ 200 nm	–	–	Dye degradation	High pressure mercury lamp (100 W, 365 nm) and ultrasonic mechanical vibrations (~ 40 kHz, ~ 5.05 × 10^4^ kPa)	Degradation ratio of 90% for methyl orange solutions (100 mL, 5 mg/L) within 120 min	[109]
ZnO	Hydrothermal reaction	No poling	Nanorods	Diameter: ~ 25 nm; length: ~ 1.25 μm	–	–	Dye degradation	Ultrasonic mechanical vibrations	Degradation ratio of ~ 80% for acid orange 7 solutions (50 mL, 5 μM) within 50 min	[184]
ZnO–PDMS	Gas-phase method	No poling	Tetrapod structure	Leg length: ~ 10 μm	22.5 pm/V	–	Dye degradation	Ultrasonic mechanical vibrations (40 kHz, 400 W)	Degradation ratio of ~ 94% for rhodamine B solutions (40 mL, 5 mg/L) within 120 min	[37]
MoSe_2_	Hydrothermal reaction	No poling	Nanoflowers	2–3 μm	–	–	Dye degradation	Ultrasonic mechanical vibrations (40 kHz, 250 W)	Degradation ratio of ~ 90% for rhodamine B solutions (50 mL, 10 ppm) within 30 s	[81]
MoS_2_	Hydrothermal reaction	No poling	Nanoflowers	0.5–1 μm	–	–	Dye degradation	Ultrasonic mechanical vibrations (40 kHz, 250 W)	Degradation ratio of 93% for rhodamine B solutions within 60 s	[72]
MoS_2_/PDMS	Hydrothermal reaction	No poling	Nanoflowers	0.2–0.4 μm	–	23 V	Dye degradation	Ultrasonic mechanical vibrations (40 kHz, 250 W)	Degradation ratio of 99% for rhodamine B solutions within 90 min	[73]
							Triboelectric nanogenerator		Output voltage of 23 V for water flow rate of 20 mL/s	
PDMS/WS_2_	Hydrothermal reaction	No poling	Nanoflowers	< 1 μm	–	–	Dye degradation and antibacterial performance	Ultrasonic mechanical vibrations (40 kHz, 300 W)	Degradation ratio of 90% for rhodamine B solutions (40 mL, 10 mg/L) within 90 min	[82]
PVDF–HFP	Crystalline thermoplastic reaction	4 V, 15 h	Solid electrolyte sheet	Thickness: 4 mm	23 pC/N	–	Self-healing	A constant voltage of 4 V	A weight gain of 6–7% at anode	[186]
PVDF	Commercial obtained	Polarized	Film	Thickness: ~ 110 μm	–	~7 V	Self-charging power cell	Compressive force (2.3 Hz, 45 N)	Voltage increased from 327 to 395 mV within 240 s	[44]
CuO/PVDF	Spin-coating method	Polarized for 30 min under 20 kV/mm at 80 °C	Film	Thickness: ~ 80 μm	–	~2.8 V	Self-charging power cell	Compressive force (1 Hz, 30 N)	Voltage increased from 50 to 169 mV within 240 s	[180]
PVDF–PZT	Spin-coating method	Polarized for 30 min under 20 kV/mm at 80 °C	Film	Thickness: ~ 90 μm	500–600 pC/N	~1.3 V	Self-charging power cell	Compressive force (1.5 Hz, 10 N)	Voltage increased from 210 to 297.6 mV within 240 s	[181]
PVDF	Spin-coating method	Polarized for 30 min under 20 kV/mm at 80 °C	Mesoporous film	Pore diameter: 700–900 nm; thickness: 2.7 μm	–	2.84 V	Self-charging power cell	Compressive force (1.8 Hz, 34 N)	Voltage increased from 160 to 299 mV within 250 s	[83]
PVDF	Spin-coating method	Polarized for 2 h under 20 V/μm	Highly porous film	Pore diameter: 1–3 μm; thickness: 30–40 μm	–	3.84 V	Self-charging power cell	Compressive energy (1 Hz, 282 mJ)	Voltage increased from 1.2 to 1.4 V within 200 s	[84]
PVDF–ZnO	Solution-casting method	No poling	ZnO nanowires in a PVDF film	Length of ZnO: 3–5 μm	–	5 V	Self-charging supercapacitor power cell	Compressive force (18.8 N)	Voltage increased from 35 to 145 mV within 300 s	[45]
PVDF–PTFE	Commercial obtained	Polarized	Film	Size: 3 × 2.5 cm^2^; thickness: 110 μm	–	2.3 V	Hybrid nanogenerator	Vibration frequency of 3 Hz and temperature variation period of 200 s	Carbon steel electrodes can be protected from corrosion for 15 h	[90]
Collagen	Obtained from rabbits’ bones	No poling	–	–	–	–	Self-healing	Compression; immersed in SBF for 28 days	Appreciable deposition of hydroxyapatite	[85]

## Mechanism of Piezoelectric Controlled Electro-chemistry

### Fundamental Electro-chemical Mechanism

The piezo-electro-chemical processes, which involve the coupling of piezoelectricity and electro-chemistry, are chemical reactions driven by piezoelectrically induced electric charge and voltage. The fundamental quantitative relationships in electro-chemistry can be concluded as the well-known law of ‘Faraday’s laws of electrolysis,’ published by Faraday in 1834 [91]. During a typical electro-chemical reaction, the mass of produced material (*m*) is related to the total transferred electric charge (*Q*), which can be summarized by:

1 $$m = \frac{QM}{Fz}$$

where *M* and *z*, related to specific materials, represent the molar mass of the substance in grams per mol and the valence number of ions of the substance (individual electric charges transferred per ion), respectively. In addition, *F* is known as the Faraday constant with a fixed value of 96,485.3 C mol^−1^. For a specific electro-chemical reaction system, it can be seen that *M*, *F*, and *z* are constant, so that the larger the value of *Q* the larger the mass of produced material, *m*.

For piezo-electro-chemistry, the magnitude of *Q* is primarily a response to the charge output of the specific piezoelectric materials as a result of a change in polarization under a mechanical load. The material factors for output will be discussed in the following section.

### Piezoelectric Material Factors

Piezoelectric material factors that influence the value of *Q* can be firstly related to aspects of the most suitable structure, since materials of the same nature and different structures have a far-reaching effect on the transfer of electric charges or ions. A range of structures have been suggested as a piezo-separator for self-charging power cells. For example, the migration rate of lithium ions can be evaluated by an important parameter, the ionic conductivity, and this materials parameter in the solid state refers to the ease of ion motion in a crystal lattice. Porous nanostructured PVDF films have shown higher ionic conductivity compared to a quasi-bulk film, and the reported explanation of this phenomenon is that the pores can act as a pathway for Li ions to move across the piezo-separator solid [83, 84]. Additionally, porous structures are beneficial for a higher intake of electrolyte solution to facilitate the migration of lithium ions. Therefore, the design of piezoelectric material structures should take account of the influence in the transfer of ions and electric charges.

In addition, in order to ensure efficient ion or electric charge transfer to surrounding atoms/molecules that participate in electro-chemical oxidation–reduction processes, the selected material requires an enhancement of the specific surface area and reactivity [91]. On varying the shape and size, especially at the smaller scale, Li et al. observed a peak shift and an intensity change of the peaks for Raman spectra of ferroelectric BFO materials due to changed spin–phonon coupling and lattice distortions [92], and a shift in absorption edge for UV–Vis absorption spectra of differing BFO samples which depended on varying crystal field intensity [93]. Thus, the optical absorption properties of materials can be strongly influenced by variations in the crystal structure including shape and size and play a significant role in electro-chemical reactions affected by light illumination; the influence of illumination on a piezo-electro-chemical reaction system will be discussed in the following section. In all, there are a variety of shape- and size-controlled physical/chemical factors that are related to mass transfer, contact area, bonding interactions as well as local crystal structure change, where factors mentioned here are generally issues of morphology, and more piezoelectric-related details will be described in the following paragraphs.

The practical output performance of piezoelectric materials closely relates to piezoelectricity, a linear electromechanical coupling, which can be considered as the following equation:

2 $$P_{i} = d_{ijk} \sigma_{jk}$$

where *P*_*i*_, *d*_*ijk*_, and *σ*_*jk*_ represent the polarization vector, the piezoelectric third-rank tensor, and the stress tensor, respectively [94]. The piezoelectric third-rank tensor *d*_*ijk*_ comprises a piezoelectric matrix with typical values dependent on the specific crystalline structure. During a typical piezo-electro-chemical process, the piezoelectrically induced open-circuit voltage (*V*_*i*_) follows the rule of piezoelectricity [27]:

3 $$Vi = \frac{{d_{ijk} \sigma_{jk} w}}{{\varepsilon_{0} \varepsilon_{r} }}$$

where *w* is the material size, *ε*_0_—is the vacuum permittivity, and *ε*_r_ is the relative permittivity. In addition, *d*_*ijk*_ is always considered as the piezoelectric charge sensitivity coefficient or piezoelectric constant in the pC/N or pm/V range [95, 96]. On the basis of Eqs. (2) and (3), the piezoelectrically induced output depends on the piezoelectric constant, the permittivity, the size, and the applied stress for a typical material.

**Fig. 2:**
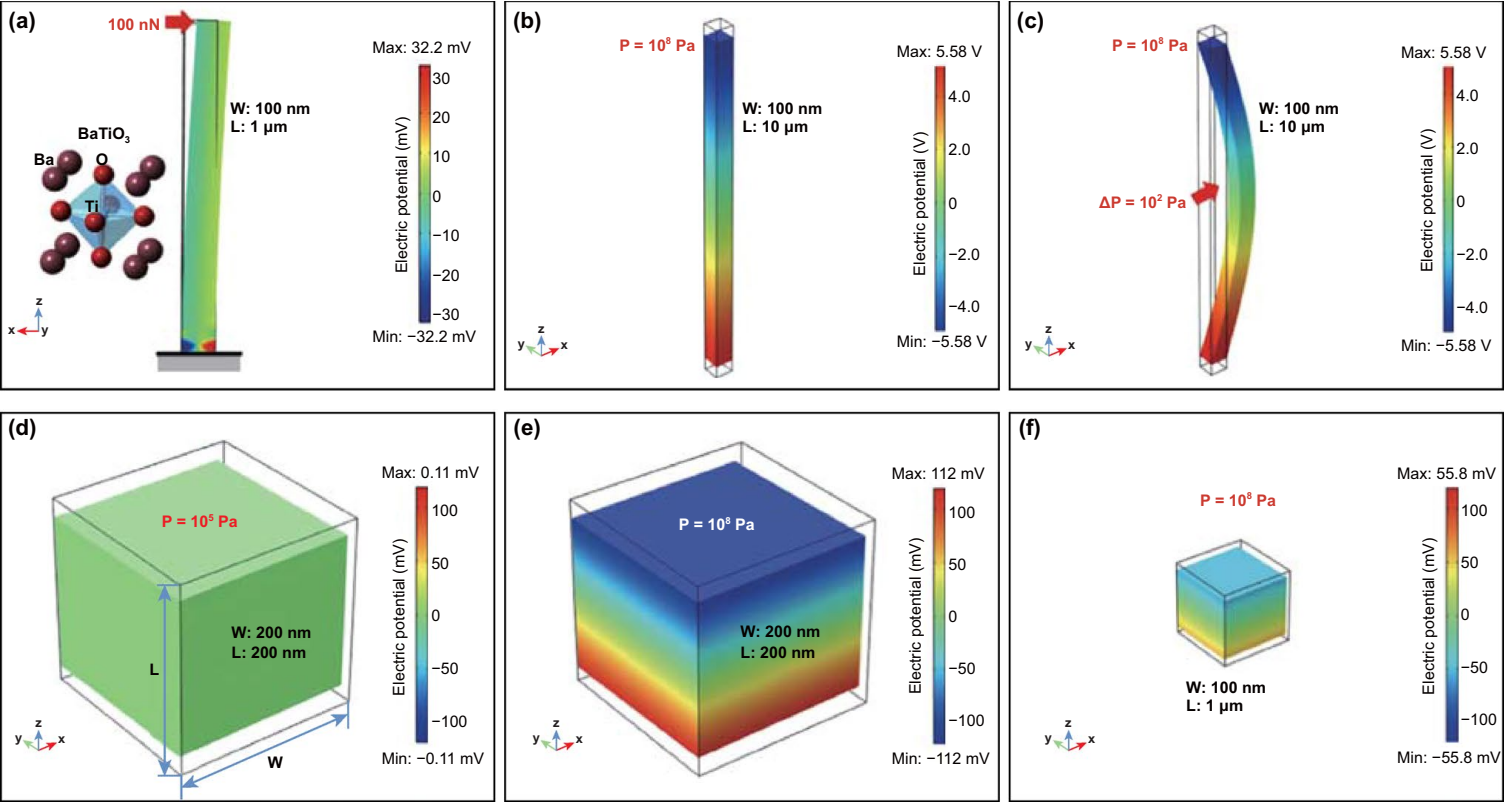
**a** A BTO nanowire with a diameter of 100 nm and a length of 1 μm stressed on the top by a lateral force with 100 nN where the bottom side is grounded and fixed. **b** A BTO nanowire under an axial compression with the pressure of 10^8^ Pa. **c** A BTO nanowire stressed at middle part under a lateral deformation with the pressure of 10^8^ Pa. A BTO nanoparticle with a size of 200 nm under the pressure of **d** 10^5^ Pa and **e** 10^8^ Pa. **f** A BTO nanoparticle with a size of 100 nm under the pressure of 10^8^ Pa. Reproduced with permission [101]. Copyright 2018, Elsevier

The loading stress for a specific material can be related to the shape of the material [78, 97–99]. A larger and easier level of deformation can be achieved in piezoelectric materials with a higher aspect ratio, which induces a higher electrical charge generation [100]. Therefore, the piezoelectric output of materials in one or two dimensions such as nanofibers (nanowires or nanorods) and nanoflowers is often larger than the equal-size spherical particles’, because of the nature of its large and easy deformation [67, 97]. Taking issues of material shape and size into consideration, the piezoelectric potential distribution of nanowires and nanoparticles was simulated via a finite element method with COMSOL multiphysics software, as shown in Fig. 2 [101]. Individual BTO nanowires oriented along the *z*-axis are strained by a point-applied lateral force, face-applied axial compression, and face-applied lateral pressure in Fig. 2a–c. For contrast, a BTO nanoparticle with a quadrilateral shape indicates the piezoelectric potential is proportional to the applied pressure and the size of nanoparticle, as in Eq. (3). When the applied pressure is 10^8^ Pa, BTO nanowire and nanoparticle of the same size of 100 nm exhibit distinctly different voltage outputs: 11.2 V for the nanowire and only 1.1 V for a nanoparticle due to higher and easier deformation of the nanowire. These simulation results are consistent with the actual phenomena of observations, where the practical degradation rate constant *k* is in the order: *k*_nanowire_ > *k*_nanoparticle_ [101].

In addition, the loading stress created by external forces or mechanical vibrations can induce a forced oscillation of the material. When an oscillating force is applied at the resonant frequency of a material/structure, it will oscillate at a higher amplitude compared to non-resonant frequencies, where a resonant frequency is in harmonic proportion to a natural frequency of material. Therefore, a larger deformation can be achieved in materials forced at its resonant frequency. A variation in the geometry, size, crystalline structure, and atomic composition can influence the fundamental mechanical properties of materials. Here, we consider materials under an elastic deformation, which obey the rule of resonance, as follows [102]:

4 $$f^{r} = \frac{x}{2l}\sqrt {\frac{E}{\rho }}.$$

Equation (4) describes the resonance frequency (*f* ^*r*^) of a typical rod vibrated longitudinally, where *l*, *E*, and *ρ* are the length, Young’s modulus, and density of the rod material, respectively. In addition, *E* is one of mechanical properties that define the relationship between stress and strain.

5 $$f^{r} = \frac{{11.2x^{2} }}{{\pi l^{2} }}\sqrt {\frac{EI}{\rho S}}.$$

Equation (5) is based on the same rod material vibrated by a lateral oscillation, where *I* and *S* represent the axial moment of inertia and cross-sectional area, respectively.

6 $$f^{r} = \frac{\pi H}{2}\left[ {\left( {\frac{x}{a}} \right)^{2} + \left( {\frac{y}{b}} \right)^{2} } \right]\sqrt {\frac{E}{{12\rho (1 - \sigma^{2} )}}}.$$

Equation (6) provides the value of *f* ^*r*^ that is dependent on a rectangular sheet with a thickness of *H* and an area size of *a* × *b*, where *σ* is Poisson’s ratio that equals to the negative of the ratio of transverse strain to axial strain. For the resonance equations above, when the values of both *x* = *y* = 1, the resonance frequency *f* ^*r*^ is the first-order resonance frequency, which can be considered as the natural frequency of the material.

According to Eqs. (4)–(6), the dependence of the resonant frequency on its vibration mode, geometry, size, and fundamental mechanical properties can be determined. A variety of piezoelectric materials show a different response to an applied mechanical force or vibration, ranging from low frequency to high frequency, so that the selection and design of piezoelectric materials or related hybrid systems can be optimized to match practical applications with a specific frequency band spectrum. As reported, the use of hydrothermally synthesized BFO square sheets for piezo-electro-chemical hydrogen production exhibits an enhanced production rate when subjected to mechanical vibrations at a frequency near their natural frequency (~ 45 kHz) compared to other frequencies [71]. For further practical applications, oceans provide a wide range of vibration energy sources with a frequency band ranging in 10 to 500 Hz for seismic exploration and commercial shipping, 500 Hz to 500 kHz for small vessel sonar, and sea-surface agitation, and > 25 kHz for thermal noise [103–107].

In this section, we have discussed the dependence of piezoelectric materials characteristics on the output performance, where the shape, size, and mechanical properties have been taken into detailed consideration. We now discuss the charge transfer mechanism for piezo-electro-chemical and piezo-photo-electro-chemical processes.

### Charge Carrier Separation and Transfer

In addition to the geometry, size, and mechanical properties of the piezoelectric materials affecting the piezoelectric output, the applied experimental conditions are also of importance for piezo-electro-chemical processes. To induce piezoelectricity, mechanical vibrations with a specific orientation and amplitude can affect the emergence of electric carriers [19, 108]. In addition, illumination by light leads to excited photo-electro-chemical reactions in piezoelectric materials that are affected by the built-in piezoelectric potential of the material [38, 109].

During a typical piezo-electro-chemical reaction, the charge transfer mechanism can be described by the following. When there is no externally applied mechanical force on the piezoelectric material, it remains at equilibrium, with occupiable electronic states and the surface energy bands in quasi-static states. When subjected to only mechanical vibrations, piezoelectricity leads to a change of polarization, which can develop a piezoelectrically induced electric field across the piezoelectric, thereby leading to electric charge carriers reorienting across different ends of the material. Consequently, both the occupiable electronic states and the surface energy bands are affected by the accumulation of these electric charges on the different sides of the material [110, 111]. Of particular note is that the temperature of the reaction system must be over 0 K; as a result, separated electron–hole pairs in such a piezoelectric semiconductor can be thermally activated. The above-mentioned occupiable electronic states and bending energy bands guide the thermally activated separated electron–hole pairs to the surface of the material, which can participate in oxidation and reduction reaction to generate active species for electro-chemical processes [37]. When the accumulated electric charges on the material surface counteract the built-in electric field, the system returns to an equilibrium level. The reverse change of polarization results in reverse accumulation of electric charges and reverse transfer of electron–hole pairs.

**Fig. 3:**
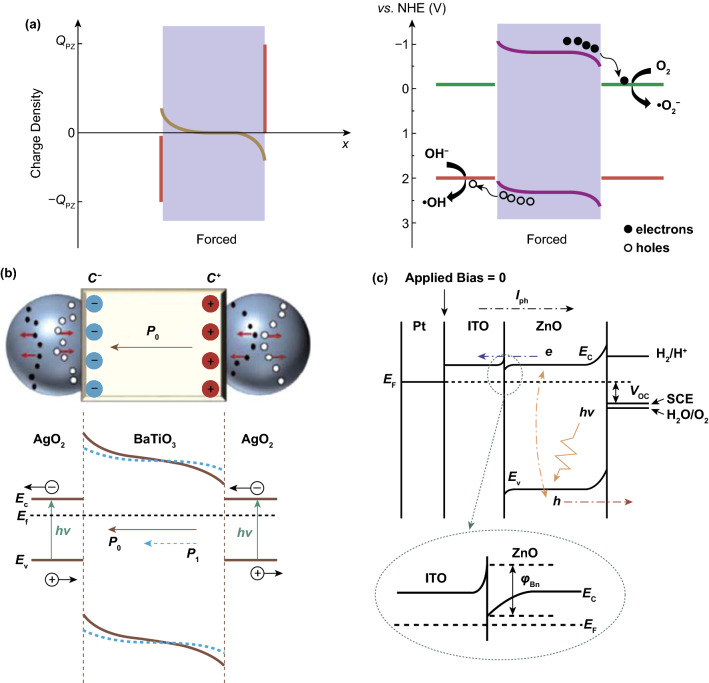
**a** Schematic of the piezo-catalytic effect in BTO–PDMS. Reproduced with permission [37]. Copyright 2019, American Chemical Society. **b** Schematic of charge carrier separation and transfer in Ag_2_O nanoparticles attached to two opposite surfaces of a BTO nanocube. Reproduced with permission [36]. Copyright 2015, American Chemical Society. **c** Schematic illustration of the band lineup of the entire PEC system. Reproduced with permission [116]. Copyright 2011, American Chemical Society

A schematic of this piezoelectrically induced charge transfer mechanism is shown in Fig. 3a, which is a system that is stimulated by mechanical vibration only [37]. The variation of the orientation and magnitude of polarization electric field across the material depends on the material type (ferroelectric or piezoelectric), and the force as a function of time since both varying the direction of vibration and mechanical intensity can influence the polarization field. Starr and Wang have pointed out the difference between the three subcategories of materials in terms of their polarization and electric dipoles [3]. In the absence of strain, ferroelectric materials exhibit a spontaneous polarization, where positive and negative electric charge centers exhibit no superposition, giving rise to resultant electric dipoles along the material, while piezoelectric materials without ferroelectricity such as quartz exhibit a zero internal dipole. However, upon the application of a strain both polarization orientation and magnitude can be varied for piezoelectric materials, since there is a separation between the positive and negative electric charge centers, where the polarization orientation is related to the direction of applied force in general.

For individual photo-electro-chemical process in a semiconductor, as is well known, the absorption activity is determined by the band gap, which is relevant to the electronic energy states [112]. For a typical semiconductor with a direct band gap which is suitable for the separation of electron–hole pairs, the general absorption coefficient near the band edge obeys the Tauc equation [93, 113]:

7 $$\alpha h\nu = A\left( {h\nu - E_{\text{g}} } \right)^{1/2}$$

where *α*, *h*, *ν*, *A*, and *E*_g_ are the absorption coefficient, Planck’s constant, irradiation frequency, proportionality constant, and energy band gap, respectively. According to Eq. (7), the adsorption activity is relevant to irradiated light and the material’s *E*_g_. Generally, irradiated light sources with a different wavelength and intensity throughout the reaction can be controlled precisely; however, the value of *E*_g_ is also related to changing spin–phonon coupling and lattice distortions which is influenced by shape and size. A high absorption coefficient is preferred for optical absorption during the photo-electro-chemical reaction and demands that the optical absorption of a material’s characteristic wavelength corresponds to the value of *E*_g_ within in the solar spectrum. In addition, the selection of material’s *E*_g_ should also take the challenge of charge separation into consideration.

In order to achieve efficient photo-electro-chemical reactions, research effort has been focused on impurity doping, increasing the reaction temperature, and the construction of heterojunction structures to restrain the recombination of photo-generated electron–hole pairs [114, 115]. When piezoelectric materials participate in photo-electro-chemical reactions, the piezoelectric polarization acts as an adjustment for the barrier height of the semiconductor at the interface, where electronic energy states can be influenced strongly. Two examples of electric charge transfer mechanism for the hybrid piezo-photo-electro-chemistry system are now described. One example is based on Ag_2_O–BTO hybrid nanostructures, where a schematic of the charge carrier transfer mechanism is shown in Fig. 3b [36]. To summarize the Ag_2_O–BTO hybrid system in brief, the Ag_2_O acts as a semiconductor to produce photo-generated carriers, while the ferroelectric polarization of BTO accelerates the separation of electron–hole pairs. Under dark conditions, there are limited electron and hole electric carriers in the Ag_2_O. When excited by photons, charge carriers generated in the Ag_2_O nanoparticles are attached on two sides of the BTO nanocube; however, electrons and holes often exhibit high rates of recombination. An electric field is built across BTO along its spontaneous polarization orientation, which provides a driving force for attracting electrons and holes moving to opposite sides, thereby reducing recombination. The separation of electron–hole pairs continues until all of the piezoelectric polarization charges are fully screened. When subjected to a compressive stress, the polarization potential is diminished, indicating the screened charges can be released, which is a fast discharge process. Subsequently, the recovery of deformation reconstructs the balance between the screened charges and the built-in field, which is a recharging process.

Another example is based on a ITO/ZnO heterojunction structure, where both ITO and ZnO are n-type semiconductors, but the free charge carrier concentration of ZnO is much lower [116]. Figure 3c demonstrates the band lineup of the photo-electro-chemical reaction system under no illumination or external bias. At the interface between ITO and ZnO, a Schottky barrier-like n–n junction is formed with a small barrier (*φ*_Bn_) due to the larger work function of ZnO. In addition, the depletion region near the ZnO is much wider than that near ITO since the free charge carrier concentration of ZnO is much lower than ITO. When subjected to illumination, photo-generated separated electron–hole pairs move through the interface between ZnO and ITO and eventually reach the electrode and electrolyte to participate in electro-chemical oxidation–reduction reactions. Therefore, the heterojunction barrier *φ*_Bn_ between piezoelectric semiconductor and electrode becomes a significant obstacle for charge transfer. Additionally, when a piezoelectric semiconductor ZnO is subjected to a tensile strain, the energy bands near the electrolyte increase, where the valence band (VB) is closer to the oxidation potential (*E*_ox_). Thus, holes are sufficiently active to drive the oxidation process, and electrons on the conductor band (CB) drift to the ITO side due to the increase of the energy band near the electrolyte interface. Meanwhile, the energy bands of ZnO decrease near the ITO region, and *φ*_Bn_ decreases as a result that can benefit charge transfer. When the piezoelectric semiconductor ZnO is subjected to a compressive strain, the energy bands near the interface of ZnO and ITO increase, while those near the interface of ZnO and electrolyte decrease. As a result, there is an increase in *φ*_Bn_, so that the separation and transfer of the photo-generated electric charge carriers show a further restrain, which prevents the progress of the electro-chemical reactions.

### Electro-chemical Processes in Specific Applications

In the previous section, we have discussed electro-chemical processes controlled by piezoelectricity. A detailed description and contrast in mechanisms for specific applications will be provided in this section. As mentioned in Sect. 2.1, electro-chemical processes for practical applications obey Faraday’s law of electrolysis. The premise of whether or not a redox reaction can occur is the relationship between the induced piezoelectric output and the oxidation–reduction potentials of the target products. The possible electro-chemical equations in detail for a variety of applications are given in Table 2.

**Table 2:** Reported electro-chemical reactions in a variety of piezoelectric applications

Piezo-electro-chemical applications	Electro-chemical reactions
Selective deposition	Ag^+^ + e^−^ → Ag
	Sn^2+^ + 2e^−^ → Sn
	H_2_O + 2h^+^ → 1/2O_2_ + 2H^+^
	Al^3+^ + 3e^−^ → Al
	Zn^2+^ + 2e^−^ → Zn
	Pb^2+^ + 2H_2_O + 2h^+^ → PbO_2_ + 4H^+^
	PbO + 2h^+^ → Pb^2+^ + 1/2O_2_
	PbO_2_ + 2h^+^ → Pb^2+^ + O_2_
	3NO_3_^−^ + 4H^+^ + 2e^−^ → N_2_O_4_ + 2H_2_O
	NO_3_^−^ + 4H^+^ + 3e^−^ → NO + 2H_2_O
	2Cl^−^ + 2h^+^ → Cl_2_ (g)
	Cl^−^ + 4H_2_O + 8h^+^ → ClO_4_ + 8H^+^
	Cl^−^ + H_2_O + 2h^+^ → HClO + H^+^
	Cl^−^ + 2H_2_O + 4h^+^ → HClO_2_ + 3H^+^
	Fe^2+^ + 2e^−^ → Fe
Hydrogen production	2H^+^ + 2e^−^ → H_2_
	H_2_O + 2h^+^ → 2H^+^ + 1/2O_2_
Dye degradation	e^−^ + O_2_ → ·O_2_^−^
	h^+^ + OH^−^ → ·OH
Self-charging power cells	LiCoO_2_ → Li_1_ _−_ _*x*_CoO_2_ + *x*Li^+^ + *x*e^−^
	TiO_2_ + *x*Li^+^ + *x*e^−^ → Li_*x*_TiO_2_

For selective deposition, the characteristic oxidation–reduction potentials of a variety of metal salts lead to various electro-chemical reactions occurring on opposite facets of the piezoelectric particles. Typical selective deposition reactions take place on the surface of piezoelectric BTO in AgNO_3_ and Pb(C_2_H_3_O_2_) aqueous solutions, where the chemical equations are illustrated as follows [77]:

8 $${\text{Ag}}^{ + } + {\text{e}}^{ - } \, \to \,{\text{Ag}}$$

9 $${\text{Pb}}^{2 + } \, + \,2{\text{H}}_{2} {\text{O}}\, + \,2{\text{h}}^{ + } \, \to \,{\text{PbO}}_{2} \, + \,4{\text{H}}^{ + }.$$

For piezo-electro-chemical hydrogen evolution, different piezoelectric materials generate a specific voltage when subjected to applied force, and piezoelectric materials can theoretically drive electro-chemical hydrogen production when the piezoelectrically induced potential exceeds the oxidation potential of hydrogen ions (1.23 V), where the fundamental electro-chemical reactions of hydrogen production are as follows [69]:

10 $$2{\text{H}}^{ + } + 2{\text{e}}^{ - } \to {\text{H}}_{2}$$

11 $${\text{H}}_{2} {\text{O}}\, + \,2{\text{h}}^{ + } \, \to \,2{\text{H}}^{ + } + \,1/2{\text{O}}_{2}.$$

Here, the mass of produced hydrogen and oxygen is in proportion to the amount of generated electric charge, and a large amount of hydrogen production is preferred, where of great significance is the long lifetime of negative charges actively for hydrogen generation. In order to decrease the recombination of piezoelectrically induced charges and extend the lifetime of negative charges, sodium sulfite (Na_2_SO_3_) is a common sacrificial agent which can scavenge positive charges effectively [117]. For piezo-electro-chemical dye degradation, the generation of active radicals is suggested as the necessary species for further decomposing organic dye molecules, such as superoxide (·O_2_^−^) and hydroxyl (·OH) radicals [118–120]. Brief procedures for piezo-electro-chemical wastewater treatment can be expressed by the following [69]:

12 $${\text{e}}^{ - } + {\text{O}}_{2} \to {\cdot}{\text{O}}_{2}^{ - }$$

13 $${\text{h}}^{ + } \, + \,{\text{OH}}^{ - } \to \cdot {\text{OH}}$$

14 $$\cdot {\text{O}}_{2}^{ - } + \cdot {\text{OH}}\, + \,{\text{dye}}\, \to \,{\text{degradation}}\,{\text{products}}.$$

The degradation products generally include 2-naphthol, 2-hydroxy-1,4-naphthoquinone, and smaller aromatic intermediates [87, 121–123].

**Fig. 4:**
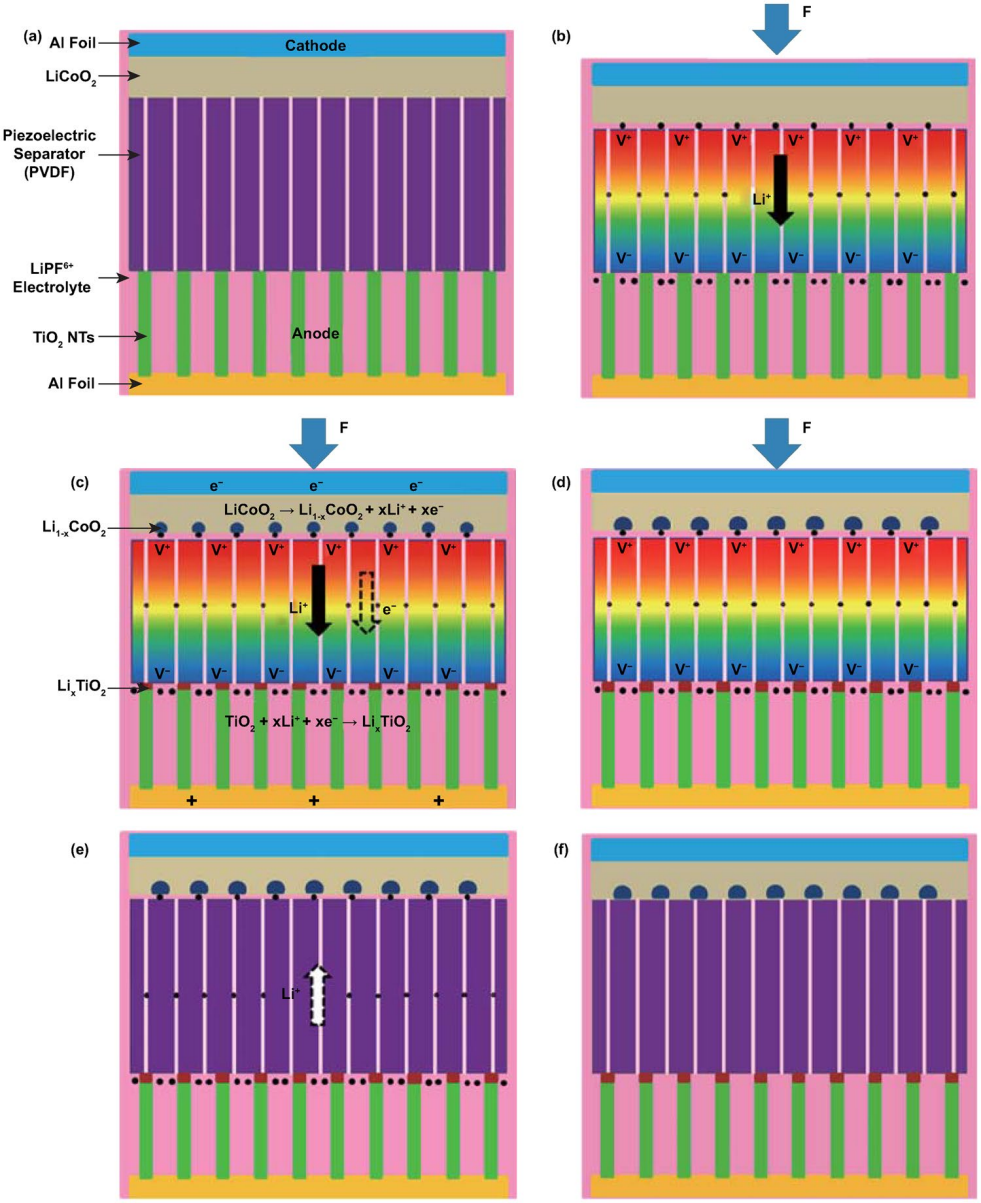
**a** Schematic of the self-charging power cell in discharged state. **b** Under compressed stress, piezoelectric PVDF film can create a potential. **c** Under the piezoelectric field, Li ions are driven to migrate from the cathode toward the anode, which leads to the corresponding charging reactions at the electrodes. **d** Chemical equilibrium is rebuilt in the self-charging power cell. **e** When the compressive stress is released, the piezoelectric field across the piezo-separator disappears; in the meanwhile, the Li ions diffuse to the cathode side. **f** New chemical equilibrium is reached, and one typical cycle of self-charging reaction is accomplished. Reproduced with permission [44]. Copyright 2012, American Chemical Society

The electro-chemical processes in self-charging power cells exhibit a more complex behavior, which can be generally divided into several steps for charging reactions driven by a cyclic compressive strain. If we take a typical self-charging power cell device as an example, as shown in Fig. 4, when the power cell device is subjected to compressive strain, the piezoelectric PVDF separator builds positive and negative potentials at the cathode and anode sides, respectively. Li ions from the cathode move across the piezo-separator film driven by the built-in piezoelectric field. This process can be considered as the charging reactions for the special lithium-ion battery, where the electro-chemical oxidation–reduction reactions occur at the cathode and anode sides as follows [44]:

15 $${\text{LiCoO}}_{2} \, \to \,{\text{Li}}_{1 - x} {\text{CoO}}_{2} + x{\text{Li}}^{ + } + x{\text{e}}^{ - }$$

16 $${\text{TiO}}_{2} + x{\text{Li}}^{ + } + x{\text{e}}^{ - } \to {\text{Li}}_{x} {\text{TiO}}_{2}.$$

Meanwhile, free positive and negative charges dissipate, respectively, at the cathode and anode sides, until an electrostatic equilibrium status is rebuilt. When the compressive strain is released, partial Li ions diffuse back to the cathode, and the as-mentioned electro-chemical reactions process to the inverse sides. Thus, when a cyclic compressive strain is applied, the power cell can realize intermittent self-charging cycles. From the perspective of a chemical reaction, the greatest difference of self-charging power cells with other electro-chemical systems is that the reactions can be controlled to react toward the inverse directions when subjected to appropriate conditions.

In Sect. 2, we overview the reported mechanisms of coupling piezoelectric and electro-chemical effects. In the following section, specific piezoelectric materials applied in electro-chemical processes will be described in detail.

## Piezoelectric Materials in Electro-chemical Processes

Since Jaffe discovered lead zirconate titanate ferroelectric ceramics in 1954 [124], lead-based piezoelectric materials including ceramics as well as single crystals have been fabricated that operate in a wide variety of transducer devices (SONAR, sensors, and actuators) and occupied an essential place in functional materials for the following three decades as a result of their high performance and stable piezoelectric properties. More recently, these materials have been used as particles for the hydrogen evolution reaction and wastewater treatment in electro-chemical applications [125–129]. However, these lead-based piezoelectric materials contain the element Pb, which is one of the major heavy metal pollutants and can endanger human health [130–133]. Lead-free piezoelectric materials are being examined intensively as a green initiative [134–137], and due to the need for environmental protection and socially sustainable materials, research related to alternative lead-free materials has become a significant research activity in recent decades [130, 138, 139]. The general piezoelectric/ferroelectric perovskites (ABO_3_-type) [140, 141], in particular barium titanate [142–145] and bismuth ferrite [146–149], have been utilized to process functional advanced devices [150–153]. In addition to ABO_3_-type ferroelectric materials, wurtzite and non-ferroelectric zinc oxide materials have been considered as advanced piezoelectric materials and have been prepared in a variety of dimensions and morphologies to convert mechanical vibration energy into electric charge for a range of applications [18, 25, 47, 69, 154–156]. During the last 10 years, remarkable piezoelectric properties have been observed in two-dimensional (2D) layered transition metal dichalcogenide-based materials with single-layered and odd-layered structures [72, 82, 157, 158], such as molybdenum disulfide [159–162], tungsten disulfide [163, 164] and molybdenum diselenide [165–169], which have received worldwide scientific attention for electronic device applications and nanoscale electromechanical systems. The piezoelectric effect in 2D layered materials is a consequence of the non-centrosymmetry of monolayers [72, 157, 158]. In addition to inorganic piezoelectric materials, organic piezoelectric materials such as polyvinylidene fluoride possess the ability to convert mechanical stress into electricity, and represent the most widely available piezoelectric polymer in functional materials and devices due to their physical characteristics of transparency and mechanical flexibility [45, 84, 90, 170–175]. In addition, specific biomaterials, such as collagen, exhibit piezoelectric properties which have been linked to the promotion of healing and reconstruction, due to the polar uniaxial orientation of molecular dipoles in the structure [10, 85, 176–178].

Above all, typical piezoelectric materials can be divided into groups of piezoelectric/ferroelectric perovskites [140–149], wurtzite zinc oxide materials [18, 27, 50, 72, 157–159], layered transition metal dichalcogenide piezoelectric materials [72, 82, 157–169], organic piezoelectric materials [45, 84, 90, 170–175], and piezoelectric biomaterials [10, 88, 179–181]. For piezo-electro-chemical processes, researchers have fabricated both single-component [76, 81] and polynary systems [49, 74, 90], and piezoelectric nano-/micro-/bulk materials with a variety of shapes, sizes, and piezoelectric constants using a variety of approaches. Depending on the preparation method, the material size spans the nanoscale to macroscale and the synthesis methods include hydrothermal reactions [47, 75], sol–gel processes [77, 179], ball-milling methods [38, 49], high-temperature calcination [74], chemical precipitation [36], electrospinning [37], thermal evaporation [79], spin-coating [180, 181] and bioactive extraction [85]. Bulk polycrystalline ferroelectric materials require poling to exhibit a remnant polarization and piezoelectricity; however, a number of microscale and nanoscale materials formed by bottom-up manufacturing, whose sub-crystal grows along certain crystallographic face, exhibit piezoelectric effects without undergoing a poling process [182, 183].

Piezoelectric coefficient aside, each of materials with different morphologies has respective advantages. Piezoelectric materials with high aspect ratio are preferred to obtain optimum deformation under a mechanical load, which can realize a high piezoelectric output. In general, materials in fiber and sheet form possessing high aspect ratio are beneficial to induce high piezoelectric output. Besides, some morphologies such as flower and nanostructured surface own high specific surface area, which makes large contact area between piezoelectric materials and solution medium, and piezo-electro-chemical performance can be improved. In addition, bulk and particle materials have advantages in material preparation.

The range of materials used to control electro-chemical reactions is now discussed in detail.

### Piezoelectric/Ferroelectric Perovskites Morphologies

**Fig. 5:**
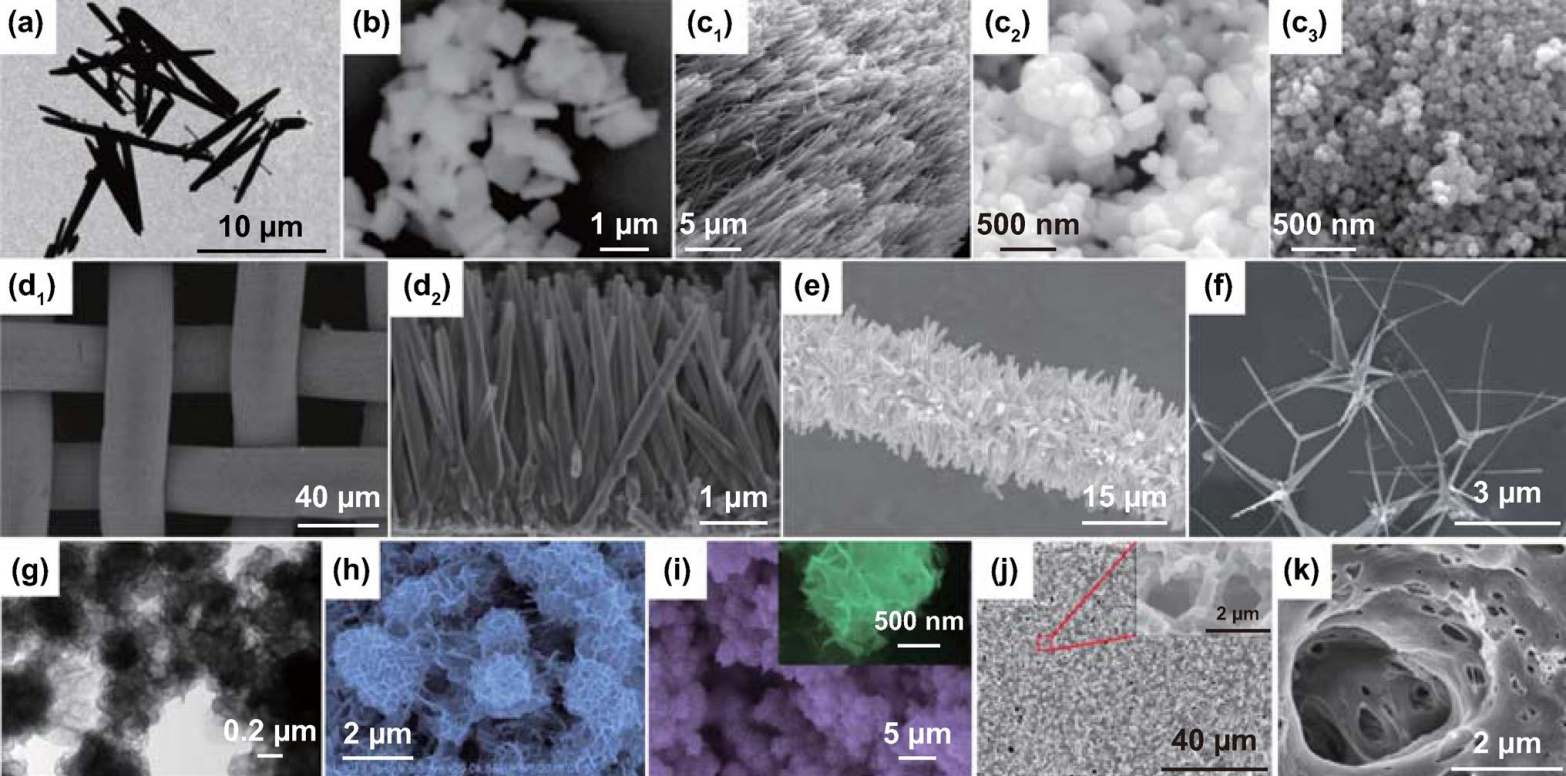
**a** TEM of PZT fibers. Reproduced with permission [67]. Copyright 2014, AIP Publishing LLC. **b** SEM image of BFO square micro-sheets. Reproduced with permission [70]. Copyright 2017, Elsevier. **c** SEM images of hydrothermal BTO nanowires, hydrothermal BTO nanoparticles, and commercial BTO nanoparticles. Reproduced with permission [101]. Copyright 2018, Elsevier. **d** SEM images of bare ZnO nanowire arrays on stainless steel mesh. Reproduced with permission [80]. Copyright 2016, American Chemical Society. **e** SEM images of ZnO nanowires. Reproduced with permission [88]. Copyright 2015, Elsevier. **f** SEM image of Ag_2_O/T-ZnO nanostructures. Reproduced with permission [79]. Copyright 2016, Royal Society of Chemistry. **g** TEM image of MoS_2_ nanoflowers. Reproduced with permission [73]. Copyright 2017, Elsevier. **h** SEM image of WS_2_ nanoflowers. Reproduced with permission [82]. Copyright 2018, Elsevier. **i** SEM image of MoSe_2_ nanoflowers. Reproduced with permission [81]. Copyright 2017, Elsevier. **j** SEM image of PVDF mesoporous nanostructured film in a top view. Reproduced with permission [83]. Copyright 2014, Elsevier. **k** PVDF surface image. Reproduced with permission [84]. Copyright 2015, Elsevier. **l** Micrographs of propidium iodide fluorescent staining cells on cortical bone collagen. The nuclei of the cells are stained in red. The deformed internal side corresponds to the face subject to compression. The deformed external side corresponds to the face subject to tension. Reproduced with permission [85]. Copyright 2017, Trans Tech Publications

Piezoelectric Pb(Mg_1/3_Nb_2/3_)O_3_-32PbTiO_3_ (PMN–PT) single-crystal slab with the size of 24 mm × 4 mm × 0.25 mm has been used to form a piezoelectric cantilever and achieves excellent piezo-electro-chemical hydrogen production [65]. Sol–gel processed lead zirconate titanate (PZT) has been fabricated as particles with an average size in the range of 70–100 nm and films of 70 nm in thickness for selective deposition investigations of the photochemical reaction with a variety of metal salts, and the influence of Zr/Ti ratio on the preferential growth of Ag onto their surfaces [77, 179]. Hydrothermally synthesized piezoelectric PZT fibers have been fabricated which achieved an excellent acid orange 7 dye degradation under the application of ultrasonic vibrations [67]. As shown in Fig. 5a, the as-prepared PZT fibers are several micrometers in length and 500 nm in diameter [67]. You et al. reported on hydrothermally synthesized bismuth ferrite (BFO) square sheets for harvesting mechanical vibration energy for wastewater treatment and hydrogen evolution [70, 71]. The BFO square micro-sheets are shown in Fig. 5b with an average size of ~ 1 μm, whose size and shape are used to facilitate bending of the sheets and thereby obtain a strong piezoelectric effect since their dimensions are of the same magnitude as the diameter of the cavitation-induced microbubbles in solution [70]. Mushtaq and coworkers prepared BFO nanosheets and nanowires via hydrothermal reactions to decompose a rhodamine B dye solution under UV light irradiation and excitation by ultrasonic vibration [78]. The nanosheets exhibit an edge length of 2–3 μm and a thickness of approximately 150 nm, and the nanowires are ~ 30 μm in length and 200–700 nm in diameter [78]. Ferroelectric barium titanate (BTO) has been produced to form a variety of particle shapes [37, 49, 75, 86, 101], nanocubes [36], nanowires [101], microdendrites [32, 87] for dye degradation and dechlorination [36, 37, 75, 76, 87, 101], hydrogen evolution [32], selective deposition [74, 86], as well as Li-ion batteries [49]. Hydrothermally synthesized BTO microdendrites researched by Hong et al. displayed a dendritic morphology with 10 μm primary branches and secondary branches with dimensions of a few micrometers, and achieved efficient direct water splitting to produce hydrogen and oxygen [32, 87]. Wu et al. compared the piezo-catalytic dye degradation activities of hydrothermal BTO nanowires, hydrothermal BTO nanoparticles, and commercial BTO nanoparticles [150]. As shown in Fig. 5c, the BTO nanowires are predominately straight, smooth, and elongated with an average diameter of 100 nm and are several micrometers in length [101]. Hydrothermal and commercial BTO nanoparticles generally exhibit a quadrilateral shape and spherical shape with average sizes of 200 and 50 nm, respectively [101]. Qian et al. reported BTO particles made by electrospinning with an average size of < 1 μm were encapsulated in polydimethylsiloxane (PDMS) as a porous foam for wastewater treatment [37]. In addition, hydrothermal zinc stannate (ZTO) nanowires showed excellent catalytic performance, with dimensions from a few 100 nm to dozens of micron range [68, 89].

### Wurtzite Zinc Oxide Morphologies

In general, wurtzite zinc oxide (ZnO) nanofibers, nanowires or nanorods have been synthesized via simple hydrothermal methods under appropriate conditions [109]. Piezoelectrically induced water splitting and dye wastewater decolorization has been performed using pure ZnO fibers and nanorods with an average diameter of 0.4 μm and 25 nm and length of 4–10 and 1.25 μm, respectively [32, 184]. In addition, ZnO nanowire arrays have been fabricated through a two-step hydrothermal processes, where ZnO seeds are initially deposited on a substrate and the nanowire arrays are then hydrothermally grow on it. Hong et al. aligned ZnO nanowire arrays and combined copper sulfide (CuS) on the surface, which led to a high piezo-photocatalytic efficiency for the degradation of methylene blue solutions using mechanical and solar energy [80]. Figure 5d shows detailed images of bare ZnO nanowire arrays on a stainless steel mesh with a diameter of 25 μm, and the nanowire arrays are vertically aligned on the mesh with an average diameter of 150 nm and an average length of 4 μm from a side view [80]. Xue et al. designed a tailored structure from seed-assisted hydrothermal ZnO nanowires that were vertically aligned on carbon fibers for decomposing methylene blue by coupling a piezoelectric with a semiconductor [88]. The ZnO nanowires attached on the carbon fibers are shown in Fig. 5e, which exhibit an average diameter and length of 500 nm and 6 μm, respectively [88]. Through thermal evaporation, tetrapod-ZnO nanostructures have been synthesized which consist of four needle-shaped legs with a 109^o^ angle between any two legs. Sun et al. achieved an ultrafast degradation of methylene blue solutions efficiently by co-application of mechanical and ultraviolet energy on silver oxide (Ag_2_O)/tetrapod-ZnO (T-ZnO) nanostructures [79]. The composite nanostructures were synthesized by the following method: First, the tetrapod-ZnO was mass produced by thermal evaporation, and then, Ag_2_O was loaded on the surface of the T-ZnO. Figure 5f shows that the T-ZnO are uniformly loaded with Ag_2_O nanoparticles on the surface, and the composite nanostructures have a tetrapod structure with four 4-μm needle-shaped legs and an average diameter of 200 nm [79]. Qian and coauthors encapsulated T-ZnO into PDMS to address the challenge of secondary pollution and reusable limits for dye degradation; the T-ZnO nanomaterials had an average needle length of ~ 10 μm [37].

### Morphology of Layered Transition Metal Dichalcogenide Based Materials

Single-layer and odd-layered transition metal dichalcogenide-based materials have been reported that demonstrate excellent piezoelectricity [72, 82, 157–168, 185]. Wu’s group has published a number of research articles on the use of hydrothermally synthesized layered transition metal dichalcogenide family nanoflowers for electro-chemical processes [72, 73, 81, 82]. An ultra-high piezo-catalytic degradation rate in the dark was achieved for single- and few-layered molybdenum disulfide (MoS_2_) nanoflowers [72]. The same MoS_2_ nanoflowers were also encapsulated in a PDMS film to destroy rhodamine B dye, and the MoS_2_/PDMS was combined with copper to produce a triboelectric nanogenerator (TENG) for wastewater mechanical energy harvesting [73]. PDMS/tungsten disulfide (WS_2_) composite materials have also been investigated for piezo-catalytic rhodamine B degradation, and the prepared WS_2_ nanoflowers achieved an almost complete antibacterial performance for Escherichia coli (*E. coli*) [82]. Single-layer and few-layered molybdenum diselenide (MoSe_2_) nanoflowers exhibited an ultra-high degradation rate to decolor rhodamine B by 90% within only 30 s [81]. Images of the typical morphologies of the range of nanoflowers that have been described above are shown in Fig. 5g–i [73, 81]. While the size of the nanoflowers exhibits some variation, a common aspect is that all possess an abundant surface area, where the MoS_2_, WS_2_ and MoSe_2_ nanoflowers are in the range of 0.4–0.6, 1–2, and 2–3 μm, respectively [72, 73, 81, 82].

### Morphology of Organic Piezoelectric Materials

Transparent and flexible organic piezoelectric polyvinylidene fluoride (PVDF) has been investigated as the most widely available piezoelectric polymer for practical electro-chemical related energy conversion applications [48, 87, 126, 173–178], which can be produced via simple spin-coating methods [45, 84, 90]. The majority of applications for PVDF-based materials have been related to battery power cells. Soroushian and coauthors proposed a poly(vinylidene fluoride-co-hexafluoropropylene) PVDF–HFP, solid electrolyte self-healing structure with 4 mm thickness and 25 mm side dimension, which was able to redistribute the structural mass in response to dynamic loads [186]. Xue et al. replaced the polyethylene (PE) separator of the battery cell with a commercial PVDF film (a thickness of ~ 110 μm) in lithium battery to drive the migration of Li ions to achieve a self-charging power cell [44]. Subsequently, this group combined a cupric oxide (CuO) anode with PVDF to create a CuO/PVDF nanocomposite anode with a thickness of ~ 80 μm and achieved stability and efficiency for the application of self-charging power cells [180]. Zhang and coworkers also designed a PVDF–PZT nanocomposite film with a thickness of 90 μm that served as a piezo-separator in self-charging power cell [181]. Xing et al. and Kim et al. reported on porous piezoelectric PVDF films to enhance the properties of self-charging power cells, respectively [83, 84]. The former processed the PVDF mesoporous nanostructured film using ZnO nanowire arrays as a template [83]. The mesopores of the PVDF share the same shape and size with hexagonal ZnO nanowire template, and the diameters of the pores ranged from 700 to 900 nm, as shown in Fig. 5j [83]. In Kim’s research, an image of the mesoporous PVDF is shown in Fig. 5k, where randomly distributed and highly interconnected pores are located within the PVDF [84]. Ramadoss et al. investigated PVDF–ZnO film that acted as a separator to manufacture a self-charging supercapacitor power cell [45]. Zhang et al. verified the feasibility of PVDF films for self-powered cathodic protection nanogenerators [90].

### Morphology of Piezoelectric Biomaterials

Collagen, extracted from bone of animals, is of interest to promote the self-healing of bone due to the polar uniaxial orientation of molecular dipoles in the structure [10, 176, 177]. Karem et al. have described the electro-chemical influence of collagen piezoelectric effect in bone healing [85]. Cells on cortical bone collagen are stained by propidium iodide fluorescent, and the nuclei of the cells are red in color. The deformed internal and external sides correspond to the faces subject to compression and tension, respectively. [85]. In addition, piezoelectricity can also be found in different parts of living body, such as deoxyribonucleic acids (DNA), cartilage, tendon, dentin, ligaments, skin, as well as cell membranes, which plays a significant role in physiological phenomena for the living body [187–189]. However, reports on the combination of these piezoelectric biomaterials with electro-chemical processes remain limited to date, where they are focused only on the threshold of the hybrid piezo-electro-chemistry systems in piezoelectric biomaterials.

The morphologies of the variety of piezoelectric and ferroelectric materials have been described, which indicate the range of dimensions, shapes and porosity levels of the materials employed; these are summarized in Table 1. We will see later that such factors can strongly influence the surface area and resonant frequency of the particles in response to ultrasonic vibrations. The following section will overview the range of piezo-electro-chemical processes which have been explored.

## Piezo-Electro-chemical Processes and Practical Applications

An electro-chemical reaction is regarded as a chemical reaction driven by an externally supplied electric circuit [28–30]. Generally, the reaction can be classified as an electro-chemical oxidation–reduction reaction if a chemical reaction is a result of an externally supplied current, and positive and negative electrical charges are transferred between atoms or molecules throughout the whole reaction [190, 191]. Piezoelectrically induced electric fields have been used as an external electric potential for electro-chemical processes [54–56]. In this section, we will examine recent research on piezo-electro-chemistry and consider their practical applications which include selective deposition [38, 77, 86], water splitting for hydrogen production [32, 65, 69], catalytic-related dye degradation and dechlorination [76, 79, 90, 124, 125], self-charging power cells [44, 45, 49, 83], and others [50, 126].

### Selective Deposition

The depolarization field is partially internally screened for a ferroelectric material [192], and the valence and conduction band edges near the material surface can be bent by polarization charges as well as surface states due to dangling bonds and defects, where the pinning of the Fermi level is influenced by the density of electric charges [193, 194]. As a result, the electronic states can be influenced by the polarization field along the ferroelectric material, which provides an opportunity to control the atomic deposition and the growth of new material on the surface of a material [38]. The polarization of the ferroelectric materials plays an important role in controlling the deposition reaction rate and the location of material growth [38, 74, 77, 86, 179].

**Fig. 6:**
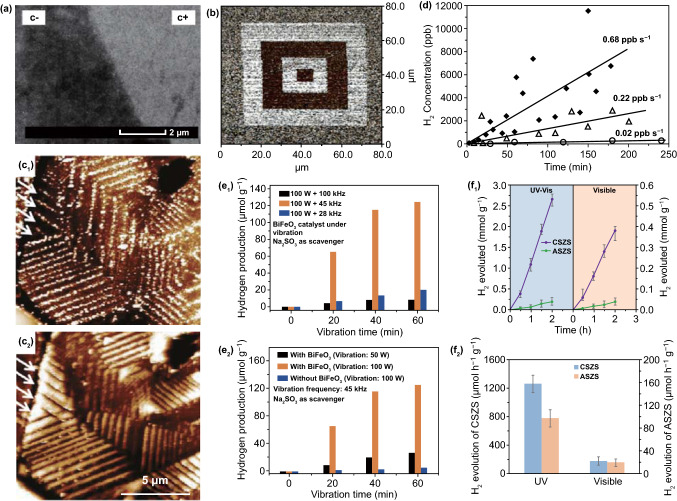
**a** SEM image of Sn deposited on the *c*^+^ domain from SnCl_2_ solution. The surface of the *c*^−^ domain was unaffected by the photoexcitation. Reproduced with permission [179]. Copyright 2008, American Chemical Society. **b** A typical PFM image of the poled pattern. Brighter squares in the picture are positive domains and the dark squares are negative domains; surrounding region is an unpoled area. Reproduced with permission [77]. Copyright 2009, Elsevier. **c** Topographic AFM images of a 15-nm-thick TiO_2_ film surface after reaction with silver nitrate solution and after reaction in lead acetate solution. Reproduced with permission [74]. Copyright 2010, American Chemical Society. **d** H_2_ concentration measured as a function of oscillating time of a piezoelectric beam in deionized water at a frequency of 10 Hz (triangles) and 20 Hz (diamonds). A Si cantilever with identical configuration was used as a control (circles). Reproduced with permission [65]. Copyright 2008, Wiley-VCH. **e** The piezo-catalytic hydrogen production from water splitting under vibration by BFO nanosheets at different vibration frequencies, and with different mechanical powers. Reproduced with permission [71]. Copyright 2019, Wiley-VCH. **f** Rate coefficients of the BHS in degradation of MO and corresponding H_2_ evolution rates. Reproduced with permission [69]. Copyright 2015, American Chemical Society

Ferroelectric PZT films have been investigated to assist in photo-induced oxidation–reduction deposition [77, 179]. Under irradiation by a 400 W Hg lamp, clear selective electro-chemical reactions could be observed on the surfaces of ferroelectric positive (*c*^+^) and negative (*c*^−^) domains, where the surface of the *c*^−^ domain was unaffected by the photoexcitation in stannous chloride (SnCl_2_) solution, which is shown in Fig. 6a [179]. In addition, the films could be polarized by an atomic force microscope (AFM) under a potential of 14 V and the shape of the deposition structure changed as a result. A piezo-response force microscopy (PFM) image of poled patterns is shown in Fig. 6b, where bright squares and dark squares represent *c*^+^ domains and *c*^−^ domains, respectively, and the surrounding area represents an unpoled region [77]. Rohrer’s group proposed to deposit titanium dioxide (TiO_2_) films on the surface of ferroelectric BFO and BTO using pulsed laser deposition [38, 74]. Spatially, the patterns of products (Ag and Pb^2+^) on the surface of TiO_2_ film reproduced those on the bare ferroelectric materials, which could be observed by AFM. As the thickness of TiO_2_ film increased, the orientation of ferroelectric dipoles had a diminished influence of selective electro-chemical reactions at the surface of the TiO_2_ film, where silver ions (Ag^+^) were reduced to silver atoms (Ag) and bivalent lead ions (Pb^2+^) were oxidized to tetravalent lead ions (Pb^4+^) under ultraviolet illumination. There are multi-domain BTO substrates with a 15-nm-thick TiO_2_ coating after selective deposition in an aqueous silver nitrate (AgNO_3_) solution and lead acetate ((CH_3_COO)_2_Pb) solution, respectively, where the arrows on the upper left corner of the figures highlight the width of the reduced Ag and oxidized Pb stripes [74].

In general, a variety of orientations of spontaneous polarization are present in bulk ferroelectric materials, while nano-/micro-piezoelectric particles can exhibit more simplex dipole arrangement due to single polarization orientation throughout the whole crystal with the sub-crystals along a certain crystallographic face [86, 182, 183]. The occupied and unoccupied energy states at the interfaces between the material and its surroundings are influenced by the spontaneous polarization, which control the movement of electrical charge. In this case, piezo-electro-chemical selective deposition can have negative effects on precisely engineered nano-/microstructures. Giocondi et al. utilized the spontaneous polarization within 1–5-μm BTO microparticles to drive photo-deposition reactions in AgNO_3_ and (CH_3_COO)_2_Pb solutions [86]. The spatial distribution of deposited silver is related to the domain structure as well as the surface orientation. The relative reactivity of the faces increases in the order of {110} < {111} < {100} [86]. In some cases, reduced silver could be exposed merely on the faces of {100}. These reactive regions were surrounded by unreactive regions in the boundaries of the face, which indicates a range of polarized domains were formed to minimize electrostatic energy.

Moreover, the above-mentioned selective deposition reactions involve semiconductive photovoltaic effects, since PZT, BTO, and BFO materials can be considered as piezoelectric/ferroelectric materials and semiconductors. The structure of the energy band can be significantly affected by the spontaneous polarization field across the semiconductors. For typical photo-electro-chemical deposition, the substances that can be deposited possess appropriate redox potentials, which must be less than the bandgap (*E*_g_) of the semiconductor. Specifically, the photo-generated electrons and holes ideally arrive at the material surface with potentials determined by the energy of the conduction band (*E*_c_) and the valence band (*E*_v_) at the surface [195]. Generally, the ability of electrons and holes to drive electro-chemical reactions is dictated by the quasi-Fermi energy of the electrons (*E*_Fn_) and holes (*E*_Fp_) at the surface, and *E*_Fn_ must be at a more negative potential than the desired reduction reaction and *E*_Fp_ must be more positive than the oxidation reaction [196, 197]. However, there is a difference in piezoelectric/ferroelectric materials since energy band tilting under an induced piezoelectric field can make the conduction band and valence band more positive or negative than the redox potentials, which determines whether the photo-electro-chemical oxidation–reduction reactions take place or not [71].

To sum up, the polarization field induced by ferroelectric materials influences the electronic states, and the polarization field is a mostly important determiner to control the selective atomic deposition, where high piezoelectric constant of materials is preferred.

### Hydrogen Production

Environmental pollution and the energy crisis are major challenges to humanity on the Earth; thus, pollution-free clean and renewable energy sources have gained increasing attention throughout the world [198, 199]. Hydrogen fuel contains abundant chemical energy and has become one of the most welcome environmentally friendly energy sources, since it possesses a high energy density and produces no greenhouse gases compared with traditional fossil fuels [200–203]. Therefore, hydrogen production has become a topic of intense interest over recent decades [200–203]. In general, hydrogen fuels can be harvested via the following approaches: coal gasification, partial oxidization, bacteria fermentation, electrolysis and water splitting [204, 205]. Piezo-electro-chemical water splitting is a recently examined approach to combine the piezoelectric properties and the electro-chemical redox reactions for hydrogen production.

Starr et al. investigated piezo-catalytic hydrogen production in a single-crystalline piezoelectric 68Pb(Mg_1/3_Nb_2/3_)O_3_–32PbTiO_3_ (PMN–PT) cantilever, where the piezoelectric cantilever was strained by a computer-controlled vibrator and linear actuator to control its high-frequency oscillation and strain state [65]. As shown in Fig. 6d, hydrogen production was related to the direct piezoelectric effect. Under different strained frequencies, H_2_ concentrations were measured as a function of oscillation time, with a constant piezoelectric potential of 20 V throughout the entire reaction. A linearized fit showed the rate of H_2_ concentration increased to 0.02, 0.22, and 0.68 ppb s^−1^ by oscillating at 0, 10, and 20 Hz, respectively. Zhang et al. realized an indirect piezo-electro-chemical hydrogen generation in a device composed of a piezoelectric bimorph cantilever based on PZT-5 ceramics [66]. The hydrogen evolution rate is relatively low at ~ 1.21 mmol h^−1^, and high ion concentration of NaHSO_4_ electrolyte aqueous solution benefits the piezo-electro-chemical water splitting reaction for hydrogen evolution. A high hydrogen generation rate of approximately 124.1 mmol g^−1^ was realized under 100 W mechanical vibration for BFO nanosheets within 1 h [71]. In Fig. 6e, the hydrogen production rates per unit mass of BFO nanosheets under 100 W vibration power are 20.4, 124.1, and 8.3 mmol h^−1^ at vibration frequencies of 28, 45, and 100 kHz, respectively. At a frequency of 45 kHz, the hydrogen evolution rate under a vibration power level of 50 W is lower than under a vibration power of 100 W, which is shown in Fig. 6e. Additionally, hydrogen and oxygen production via direct water splitting was realized by vibrating piezoelectric ZnO microfibers and BTO microdendrites using ultrasonic waves. In the work of Hong, rapid hydrogen and oxygen evolution was achieved at an initial rate of 3.4 × 10^−3^ and 1.7 × 10^−3^ ppm s^−1^, respectively, using ZnO microfibers as the piezo-catalyst in the first period (0–40 min), where the stoichiometric equivalence of the produced hydrogen and oxygen gases was H_2_/O_2_ = 2:1 [32]. When the externally applied ultrasonic wave vibrations were stopped, the production of hydrogen and oxygen terminated immediately with a H_2_ generation rate of < 0.0001 ppm s^−1^. For piezoelectric BTO microdendrites, it was shown that an average hydrogen evolution rate is 1.25 × 10^−2^ and 9.13 × 10^−3^ ppm s^−1^ during the first and second piezo-electro-chemical reactions, respectively, where the first period of vibration was 0–50 min, the second period was 100–150 min, and the ultrasonic vibrations were turned off during 50–100 min [32].

Novel metal–semiconductor branched heterostructures (BHS) of hybrid Cu/CuS–ZnO/ZnS (CSZS) and Ag/Ag_2_S–ZnO/ZnS (ASZS) materials have been used to fabricate a hybrid cell to harvest multiple energy sources, namely mechanical and solar energy, for hydrogen production [69]. The hydrogen production performance of ASZS BHS and CSZS BHS under both UV–Vis and visible illumination is shown in Fig. 6f, where the hydrogen generation rates are 1250 and 182 μmol h^−1^ g^−1^ for the CSZS BHS and 98.8 and 20 μmol h^−1^ g^−1^ for the ASZS BHS, respectively, under UV–Vis illumination and visible illumination. The CSZS BHS exhibited the highest activity under UV–Vis illumination, due to sulfurized CSZS BHS possessing a higher absorbance across the wavelengths of 300 to 800 nm. This approach was able to overcome the challenges in photocatalytic hydrogen production, such as inadequate power conversion, high recombination of photo-induced charges, and intermittent availability of solar illumination, due to visible light absorption, and high charge separation efficiency.

Results from hydrogen production procedures using the above-mentioned single or hybrid piezoelectric materials indicate that the challenges for generating hydrogen are to drive a potential, through the applied approaches of either single piezoelectrically induced or hybrid piezoelectric/photovoltaic coupling induced, in order to overcome the standard oxidation–reduction potential of water. This suggestion is discussed in more detail in Sect. 2, in relation to Faraday’s law of electrolysis. In summary, methods to reduce environmental pollution are attracting increasing research interest, and the next section will introduce piezo-electro-chemical processes for environmental purification in wastewater treatment.

### Dye Degradation and Dechlorination

Paper, dyeing, and textile industries produce a large amount of organic dye pollutant each year, which accelerates environmental pollution [206]. Dye degradation for wastewater treatment has attracted long-term and substantial attention for a variety of research fields, including environmental physics [207–209], chemistry [210–213], and biology [214–216]. Advanced chemical oxidation processes offer an environmentally friendly approach to decompose a range of dye molecules efficiently, and recently, scientists have placed significant effort in applying a piezoelectric built-in potential to drive electro-chemical wastewater treatment [72, 101].

**Fig. 7:**
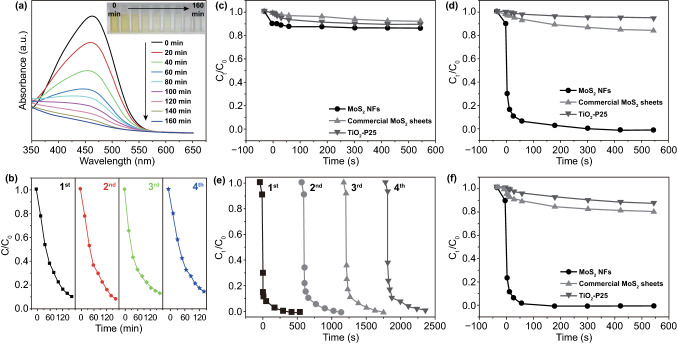
**a**, **b** UV–Vis absorption spectra of the MO aqueous solutions sampled in BTO nanowires at set intervals. Inset: photograph of the MO solutions samples; plots of the relative intensity of the maximum absorption at 460 nm as a function of reaction time in four recycling processes. Reproduced with permission [101]. Copyright 2018, Elsevier. **c**–**f** The degradation ratio of the RhB dye using the MoS_2_ nanoflowers, commercial MoS_2_, and TiO_2_–P25, under different degradation conditions: **c** in the dark, **d** ultrasonic wave in the dark, **e** the repeatable degradation tests of the RhB dye using the MoS_2_ nanoflowers under ultrasonic-wave assistance in the dark, **f** degradation ratio of the RhB dye under ultrasonic-wave assistance with the xenon lamp illumination using the MoS_2_ nanoflowers. Reproduced with permission [72]. Copyright 2017, Wiley-VCH

Piezoelectric BTO nanoparticles and nanowires were synthesized to investigate the piezo-catalytic dye degradation in aqueous methyl orange solutions, and enhanced piezo-catalytic activity was exhibited in nanowires compared to nanoparticles due to their increased susceptibility to mechanical deformation [101]. The piezo-catalytic activity of BTO nanowires for degrading methyl orange dye solutions is shown in Fig. 7a, b. The UV–Vis absorption spectra of 5 mg L^−1^ methyl orange dye solution range from 350 to 650 nm with a peak at 460 nm, where the intensity of the peak shows a sharp decrease with increasing piezo-catalytic time. Within 160 min, complete dye degradation could be achieved, as shown in the inset of Fig. 7a, where the color of the initial orange solution becomes transparent. The piezo-catalytic stability of the materials allows recycling of the BTO nanowires up to four times, while maintaining a high catalytic activity. An ultra-high degradation activity in single- and few-layered MoS_2_ nanoflowers was obtained in dark, where the degradation ratio of the rhodamine B solution was up to 93% within 60 s of the piezo-catalytic reaction [72]. In this work, the electro-chemical piezo-catalysis of the rhodamine B dye solutions in MoS_2_ nanoflowers, commercial MoS_2_, and TiO_2_–P25 has been evaluated in different conditions, as shown in Fig. 7c–f. The catalytic activities of commercial MoS_2_ and TiO_2_–P25 exhibit a relatively low performance, with or without the application of ultrasonic mechanical vibrations and light illumination. Interestingly, for single- and few-layered MoS_2_ nanoflowers, the piezo-catalytic performance increases rapidly when the ultrasonic mechanical vibrations are applied during ultrasonic excitation in the dark. The degradation ratio reached 93% within 60 s and 100% within 300 s. In general, light illumination improved piezo-catalysis and reduced the time for complete degradation to 180 s. Thus, light plays an important role in catalytic dye degradation, which is generally well known for *photocatalytic* wastewater treatment, which has been widely researched [217–219]. Research and activities of piezo-potential-modulated photocatalysis have been widely reported, where the performance of typical photocatalysis can be extended to new approaches of piezo-photocatalysis, which features the simultaneous coupling of piezoelectricity, semiconduction, photocatalysis, and photoexcitation [68, 79, 89].

**Fig. 8:**
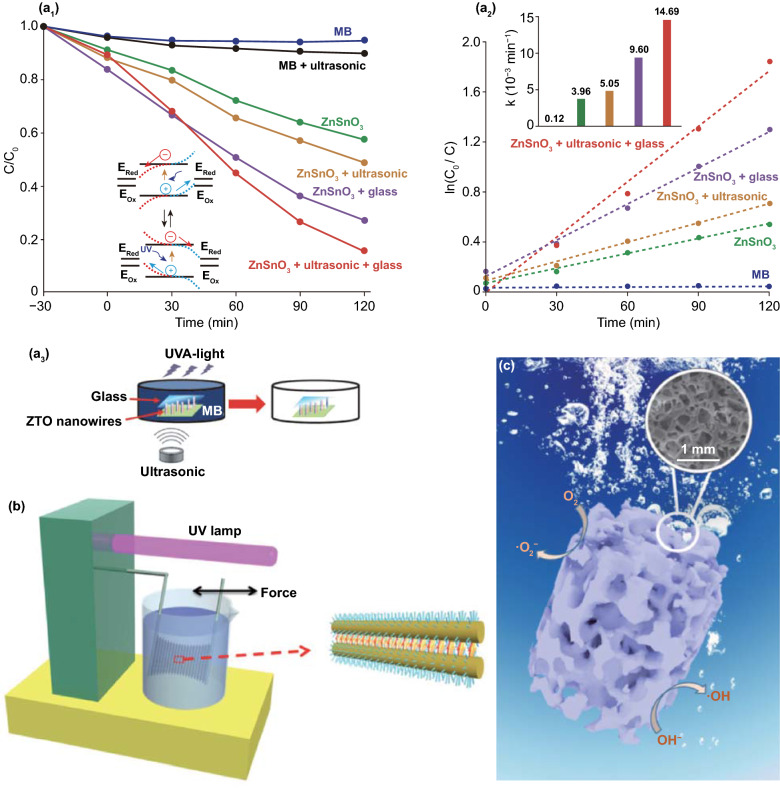
**a** Photocatalytic results of ZTO nanowire arrays under various stresses. Reproduced with permission [89]. Copyright 2016, American Ceramic Society. **b** The degradation of MB solution by the piezo-photocatalytic activity of ZnO nanowires/C fibers under UV irradiation and periodically applied force. Reproduced with permission [88]. Copyright 2015, Elsevier. **c** Schematic of porous piezo-catalysis process in BTO–PDMS porous foam. Reproduced with permission [37]. Copyright 2019, American Chemical Society

Under a combined excitation of ultrasonic mechanical vibration and UV irradiation, Ag_2_O/T-ZnO nanostructures with a mass of 2 g L^−1^ were able to completely degrade 5 mg L^−1^ methylene blue solution within 120 s [79]. The enhanced photocatalytic activity was thought to be mainly due to the piezoelectric field of the T-ZnO nanostructures and the build-in electric field of the Ag_2_O/T-ZnO heterojunctions, which can improve the separation of photo-generated positive and negative electric carriers and suppress their combination. Similarly, well-aligned LN-type single-crystalline ZTO nanowire arrays exploited synergistic piezo-photocatalysis to degrade aqueous methylene blue solutions efficiently [68, 89]. It was suggested that synergistic piezo-photocatalysis was attributed to material band bending, sono-vibration, mass transfer enhancement, as well as abundant active reaction sites. Figure 8a shows the piezo-potential-modulated photocatalytic activities of different added catalysts under a variety of applied excitations including ultraviolet A (UVA) source illumination (320–340 nm and 30 W) and ultrasonic vibration (40 kHz and 0.2 W), where ultrasonic vibration was applied with or without a 1.7-g transparent glass attached on ZTO nanowire arrays for piezo-photocatalytic measurement, and no additional pressure was applied to the reaction system for the contrastive photocatalytic measurement [68]. The self-degradation of the aqueous methylene blue solutions can be negligible, while the piezo-potential-modulated photocatalytic activity leads to an improved degradation ratio of 75% within 120 min with both ultrasonic vibration and a transparent glass applied simultaneously. When vibrations or stress are applied to the system, the ZTO nanowires are bent which lead to electric potential variations across the nanowires that enhances the movement of the electric carriers and attenuates their recombination. The photodegradation kinetic behavior of ZTO under pressure from ultrasonic vibration and a transparent glass can be calculated as a degradation rate constant (*k*) of ~ 1.5 × 10^−2^ min^−1^, which is approximately four times higher than that without any external pressure from glass or ultrasonic vibrations.

However, when piezoelectric micro-/nanocatalysts are in particulate form, they can flow into the water being treated to provide environmental challenges and secondary pollution. This can make it an expensive and time-consuming process and involve complex recycling procedures. To address the challenge of secondary pollution and reusable limits for particle based piezo-catalysis, approaches related to particle integration have been analyzed and achieved excellent piezo-catalytic activity. A two-step seed-assisted synthesized of hydrothermal ZnO nanowires led to vertically grown and tightly connected wires that were attached to polycrystalline carbon fibers, as reported by Xue and coworkers [88]. A device for both mechanical and solar energy harvesting to drive catalytic methylene blue dye degradation under UV irradiation was fabricated that was operated under a periodically applied force; this was achieved through weaving a ZnO/C composite woven using multiple fibers, as shown in Fig. 8b. Additional work on piezoelectric material encapsulation has included a composite porous foam which comprised of piezoelectric materials and a PDMS polymer which was able to generate a strong oxidizing superoxide and hydroxyl species for organic rhodamine B dye degradation under the application of ultrasonic vibrations [37], as shown in Fig. 8c. Repeated decomposition reactions were achieved for up to 12 cycles, since the porous foam could be easily placed in the reaction system and removed from wastewater.

In addition, the degradation of 4-chlorophenol and dechlorination were achieved by the mechanical deformation of tetragonal BTO (*t*-BTO) nano-/micrometer-sized particles [75]. The dechlorination efficiency of 4-chlorophenol in different catalysts is shown, and ~ 35.2% of chlorines were removed from 4-chlorophenol in *t*-BTO within 120 min. As the annealing temperature was increased, the dechlorination efficiency rapidly increases above 200 °C, due to the generation of *t*-BTO. Deposited Ag on *t*-BTO was also shown to improve the piezo-dechlorination activity.

For piezo-electro-chemical hydrogen production, dye degradation and dechlorination, the similarity of majority of these applications can be summarized that electro-chemical processes are driven by piezoelectric material under forced vibration in aqueous solution, where piezoelectric property, easy-to-bend shape, vibration frequency range, and non-toxicity of material are the notable factors for researchers.

### Self-charging Power Cells

**Fig. 9:**
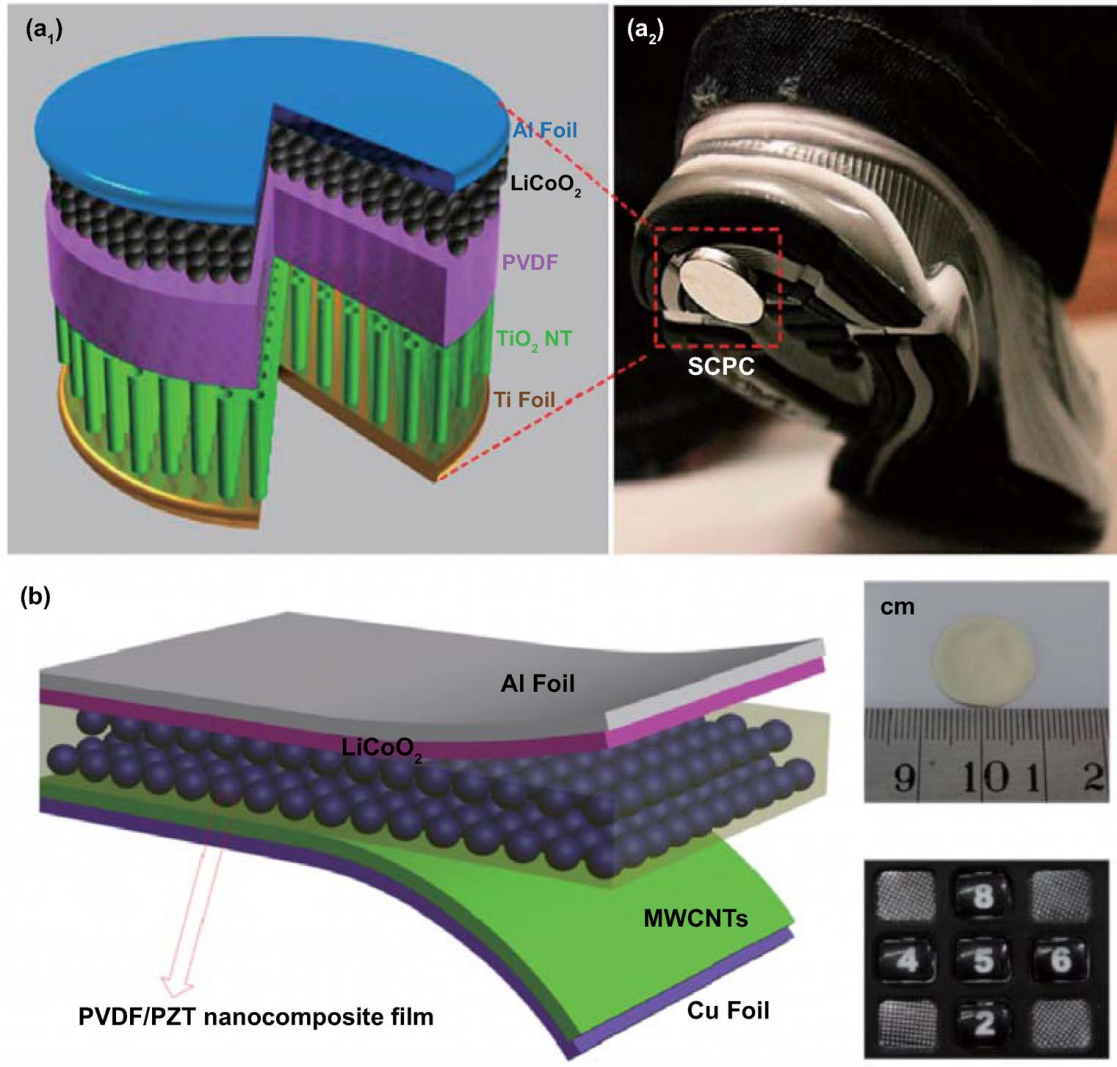
**a** Schematic showing the design and structure of the self-charging power cell. This structure is sealed in stainless steel 2016-coin-type cells, as shown in the inset. On adhering a power cell to the bottom of a shoe, the compressive stress generated by walking can be converted and stored directly by SCPC. Reproduced with permission [44]. Copyright 2012, American Chemical Society. **b** Schematic structure of a SCPC with a PVDF–PZT nanocomposite film as a piezo-separator for charge storage. Photograph of PVDF–PZT nanocomposite film. Adhering an SCPC underneath the button of a calculator, the mechanical energy generated by pressing the button can be converted and directly stored by the SCPC. Reproduced with permission [181]. Copyright 2014, IOP Publishing Ltd

Energy generation and energy storage sources are often designed as two separate unit devices where the generators can convert different forms of energy sources into electricity for energy generation and power cells can store electrical energy as chemical energy for storage, which is released on demand [220–228]. Xue and the coworkers were the first to propose an integrated self-charging power cell that hybridized both energy generation and storage [44]. As shown in Fig. 9a, the exterior appearance of the integrated self-charging power cell is that of a stainless steel coin-type cell, and the major components within the cell comprise an anode with anatase TiO_2_ nanotubes aligned on Ti foil, a cathode with LiCoO_2_/conductive carbon/binder mixtures on Al foil, and a separator formed by a polarized PVDF film. The above-mentioned structure was designed on the basis of the combination of piezoelectric effect and electro-chemistry, which can be used in practical applications of self-powered nano-/microsystems and personal electronic devices.

**Fig. 10:**
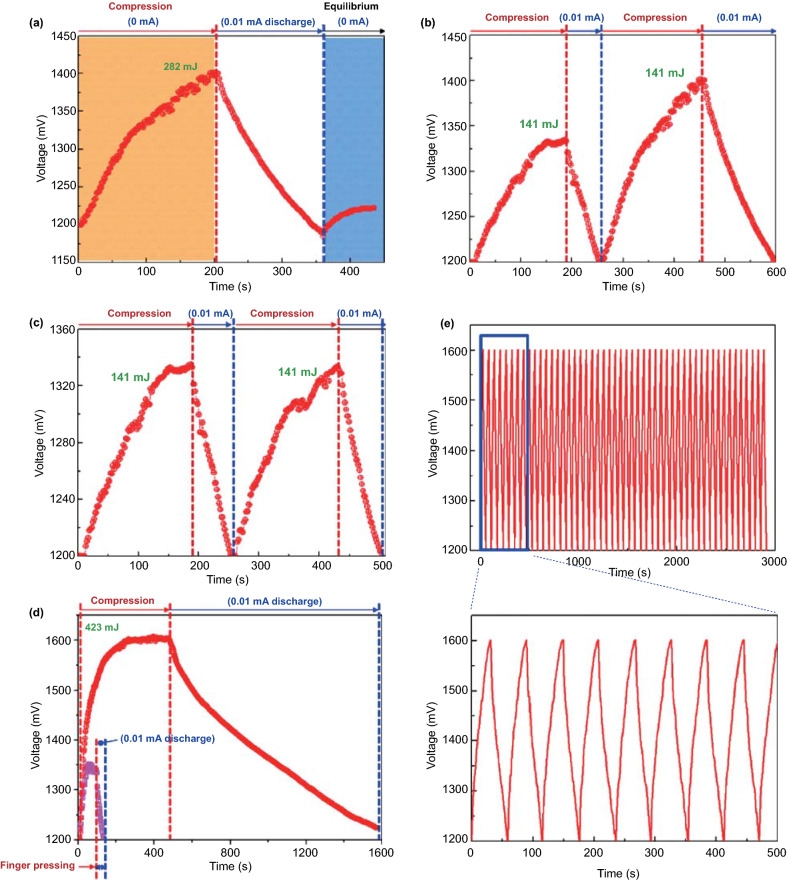
**a** Typical self-charging curve comprised by compression, discharge and equilibrium regions. **b** Self-charging cycles with different applied forces. **c** Self-charging cycles with identical applied forces. **d** Comparison of self-charging performances via compressed by finger pressure and a higher mechanical force. **e** Galvanostatic charge/discharge profile. Reproduced with permission [84]. Copyright 2015, Elsevier

In further research, the components of the electrode and separator were modified to attain a high energy conversion, high piezo-electro-chemical charge/discharge performance and high stability. The hybrid structure of the electrode on the anode side was improved and obtained high stability and efficiency, by being fabricated using a CuO/PVDF nanocomposite piezo-anode [180]. This type of piezo-anode structure benefited from an effective use of induced piezoelectric field by external mechanical deformation or vibration due to large interface area and intimate contact between the CuO and PVDF. Subsequently, the porous structure of the PVDF separation was used to achieve effective lithium ion transfer channels [83, 84], which could be attributed to a geometrical strain confinement effect. A PVDF–PZT nanocomposite film was proposed that acted as the piezo-separator to enhance the piezoelectric signals, since PZT was a more piezoelectrically active material [181]. Figure 9b shows the self-charging power cell fabricated by hybrid Al/LiCoO_2_/PVDF–PZT/MWCNT/Cu, which was constructed as a sealed stainless steel 2016-coin-type cell with a diameter of 20 mm and was positioned underneath the touch buttons of a keyboard to harvest and store mechanical percussive forces. Generally, a unidirectional force was applied on the flexible devices to create additional forces in different directions and the induced strain along different directions can be effectively improved when pores are present in the flexible materials [229]. In this case, the porous PVDF nanostructured film exhibited an enhanced piezoelectric output under an applied stress [83, 84]. The charge and discharge properties of highly porous piezoelectric PVDF membrane for a self-charging power cell are illustrated in Fig. 10 [84]. Under the application of a mechanical compression with an energy of 282 mJ and a frequency of 1 Hz, the voltage increases from 1.2 to 1.4 V within 200 s. Subsequently, the galvanostatically discharged reaction performs a constant voltage drop until 1.185 V with a current of 0.01 mA, where 0.4 μAh is needed to return to the original potential of 1.2 V. Finally, the self-charging power cell reaches an equilibrium level. Under mechanical energies of 141 and 282 mJ at a frequency of 1 Hz for 200 s, the cell can be self-charged to 1335 and 1400 mV, respectively. The charge and discharge processes are stable under the same condition and even under the application of finger pressure, the power cell could be self-charged. After mechanically shocking for 3000 s, the device exhibited no unexpected fluctuations, thereby indicating the stability of the piezo-separator in the self-charging power cell.

Supercapacitors are universally known as one of the most potential next-generation energy storage candidates because of their ultra-high power density, high charge, and discharge rates, as well as long service life [230–233]. Ramadoss et al. investigated a self-charging supercapacitor power cell with PVDF–ZnO film as the separator and MnO_2_ nanowires as the positive and negative electrodes in a supercapacitor [45]. The cell exhibited a self-charging capability under palm impact, where the aluminum-foil-based and the fabric-based devices were charged 110 and 45 mV within 300 s, respectively. In addition, green LEDs could be illuminated when the power cell acted as a power source. The application of piezo-electro-chemical-related self-charging power cells offers a promising research direction for advanced functional self-powered small-scale devices.

Piezoelectric properties aside, materials that facilitate ion transfer and flexibility are ideal issues for piezo-electro-chemical practical applications in self-charging power cells.

### Additional Piezoelectric Electro-chemical Applications

**Fig. 11:**
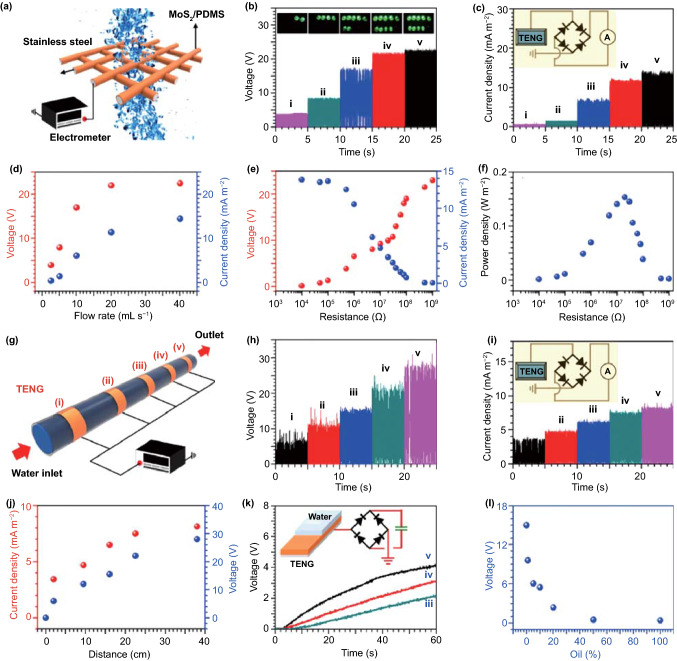
**a**–**f** Illustration and performance of nanogenerator manufactured from a MoS_2_/PDMS nanocomposite coating on a commercial stainless steel grid. **g**–**l** Illustration and performance of a thin water–TENG attached to different positions inside a polymer tube. Reproduced with permission [73]. Copyright 2017, Elsevier

In the previous section, we have discussed self-charging power cells, which combined a Li-ion battery structure and a nanogenerator for simultaneous generation and storage of energy sources. Individual nanogenerators have also been utilized in practical applications in electro-chemistry systems. Zhang et al. produced a flexible hybrid nanogenerator for cathodic protection without the need for any external power source, where the nanogenerator output protected the metal surface from chemical corrosion [90]. In Lin’s work, a MoS_2_/PDMS composite nanogenerator with a different structure exhibited sensitive characteristics which depended on the water flow rate and position, as well as contamination detection in wastewater [73]; schematics of the devices and their performance are illustrated in Fig. 11. A nanogenerator was produced by coating a MoS_2_/PDMS composite (additive MoS_2_ amount of 5% in weight percentage) on a commercial stainless steel grid, as shown in Fig. 11a–f, with the composite nanogenerator being 5 × 5 cm^2^, and the grid diameter and area of 200 µm and ~ 2 cm^2^, respectively. The nanogenerator was placed under a kitchen faucet, and the distance between the faucet and the nanogenerator was fixed at ~ 30 cm. It was observed that a faster water flow rate led to a higher output voltage and current density of the device. When the water flow rates were 2.5 and 20 mL s^−1^, the generated electric output ~ 23 and ~ 4 V was able to drive two and nine LEDs, respectively. A nanogenerator was also produced by coating a MoS_2_/PDMS composite on a flexible substrate with a conducting layer, as shown in Fig. 11g–l. The output generated was at a maximum when the nanogenerator was the nearest to the water inlet. With an increase in the distance from the water inlet, both the output voltage and current density of the generator gradually decreased. In addition, the generated output was dependent on the oil content of the water, since any interaction between the nanogenerator and oil weakened the contact between the aqueous solution and the piezoelectric nanogenerator.

Biological systems have also been examined. The self-healing phenomenon of organisms is a stable and balanced mechanism to achieve self-recovery and self-regulation [234–237]. Generally, osteoblasts control the reaction of bone healing and growth in living bodies [238]. Without osteoblasts present, Karem et al. reported that deformed collagen produced the deposition of hydroxyapatite (HA) via an electro-chemical process due to the piezoelectric effect, where the rate of deposition of HA was able to control bone healing and growth, which indicated the possibility to help with the self-healing of bones via harvesting biomechanical motion [85]. For bionic applications, self-healing devices and structures have been fabricated to re-establish the structural mass under dynamic service loads excitation via imitating biological bone healing structures. Soroushian and coauthors proposed a PVDF–HFP solid electrolyte and self-healing structure, which was able to redistribute the structural mass in response to dynamic loads due to the piezoelectricity to convert mechanical energy into electrical energy [186].

## Conclusion

This review has covered in detail current progress on the variety of piezoelectric materials for controlling electro-chemical processes to inspire rising efforts in the emerging research field. Table 1 provides a summary of the piezoelectric material types, their characteristics, and piezo-electro-chemical processes which cover a wide range of piezoelectric materials including piezoelectric perovskites, wurtzite zinc oxide, two-dimensional layered transition metal dichalcogenide family, organic piezoelectric materials, and biological materials. The material magnitude and morphology are varied for the creation of macro-/micro-/nanomaterials and include materials in bulk, fiber, sheet, flower, particle, and irregular form, which have been synthesized by different physical, chemical, or biological methods. The practical electro-chemical applications of these piezoelectric materials include selective deposition, hydrogen production, dye degradation, and self-charging power cells. The degree by which the piezoelectric materials and external conditions influence the electro-chemical system is of significant interest and has also been summarized. In order to achieve a high piezoelectric output, materials with high aspect ratio are preferred to obtain optimum deformation under a mechanical load. In addition, piezoelectric materials with a specific resonant frequency can be exploited to harvest mechanical vibrations ranging in different frequency bands for different practical applications. These factors provide a reliable guidance on the selection of piezoelectric materials for experimental designs.

Today, energy generation and environmental pollution have become significant challenges for humanity. Piezoelectric materials in electro-chemical applications can play important roles in bringing new ideas and approaches to resolve issues from challenges of energy and environment. We have seen that specific piezoelectric domains can affect the electro-chemical selective deposition, where polarization structures can be controlled by an electric field. This research can lead to the potential in developing methods to coat materials with specific polarized piezoelectric materials that can be utilized in corrosion resistance. Furthermore, the strong ability of piezoelectric materials in cleaning wastewater could be a replacement for photo-cleaning and enable piezoelectric materials to be used for self-cleaning the surface of buildings. For energy harvesting, piezoelectric materials allow the conversion of environmental mechanical vibrations across a large range of frequencies into an electric energy source. Energy storage is the capture of electric energy produced that is available for use at a later time, and self-charging power cells that use a piezo-separator can realize a simultaneous approach to combining energy harvesting and storage, which can be applied in ocean wave vibration energy harvesting and storage for further because there are abundant water fluctuations in ocean. However, piezo-electro-chemical applications in laboratory work are far inferior to commercial market to benefit humans. First, energy conversion efficiency is always needed to improve in order to achieve maximum energy use and minimize waste at sources. Second, actually most materials and devices in laboratory perform great in ideal circumstances, but when these products come to real tests, they will be difficult to maintain a long lifetime. Third, a variety of samples manufactured with sophisticated equipment and costed costly manpower and material resources in order to pursue high performance; however, cost is an indispensable consideration in business models.

In addition, the potential for coupling multiple systems is of great interest and piezo-electro-chemistry has the potential to work together with photo-electro-chemistry, including piezoelectric affected photo-deposition, photocatalytic hydrogen production, and photodegradation. To achieve piezoelectric and photovoltaic coupling, ferroelectric semiconductors can exhibit piezoelectricity, pyroelectricity, ferroelectricity, as well as photovoltaic effects, which can achieve multiple coupling in a single material. On the basis of piezoelectric, pyroelectric photovoltaic, thermoelectric, triboelectric, and flexoelectric effects, the design for their efficient coupling could be analyzed for further. Finally, although piezo-electro-chemical applications have attracted a large amount of interest with a growing number of researches, patents, and publications, the business conversion of these potential applications is necessary.

## References

[1] Curie P, Curie J (1880). Development, via compression, of electric polarization in hemihedral crystals with inclined faces. Bull. Soc. Minerol. Fr..

[2] Liang Z, Yan CF, Rtimi S, Bandara J (2019). Piezoelectric materials for catalytic/photocatalytic removal of pollutants: recent advances and outlook. Appl. Catal. B.

[3] Starr MB, Wang XD (2015). Coupling of piezoelectric effect with electrochemical processes. Nano Energy.

[4] Dahiya RS, Adami A, Collini C, Lorenzelli L (2013). POSFET tactile sensing arrays using CMOS technology. Sens. Actuat. A.

[5] Bowen CR, Kim HA, Weaver PM, Dunn S (2014). Piezoelectric and ferroelectric materials and structures for energy harvesting applications. Energy Environ. Sci..

[6] Damjanovic D (1998). Ferroelectric, dielectric and piezoelectric properties of ferroelectric thin films and ceramics. Rep. Prog. Phys..

[7] Wu J, Wu Z, Qian WQ, Jia YM, Wang Y, Luo HS (2016). lectric-field-treatment-induced enhancement of photoluminescence in Er^3+^-doped (Ba_0.95_Sr_0.05_)(Zr_0.1_Ti_0.9_)O_3_ piezoelectric ceramic. Mater. Lett..

[8] Shi PP, Tang YY, Li PF, Liao WQ, Wang ZX, Ye Q, Xiong RG (2016). Symmetry breaking in molecular ferroelectrics. Chem. Soc. Rev..

[9] Kholkin AL, Pertsev NA, Goltsev AV, Safari A, Akdogan EK (2008). Piezoelectricity and crystal symmetry. Piezoelectric and Acoustic Materials for Transducer Applications.

[10] Marino AA, Becker RO (1970). Piezoelectric effect and growth control in bone. Nature.

[11] Katsouras I, Asadi K, Li MY, van Driel TB, Kjaer KS (2016). The negative piezoelectric effect of the ferroelectric polymer poly(vinylidene fluoride). Nat. Mater..

[12] Brody PS (1973). Large polarization-dependent photovoltages in ceramic BaTiO_3_ + 5 wt% CaTiO_3_. Solid State Commun..

[13] Brody PS (1975). High-voltage photovoltaic effect in barium-titanate and lead titanate lead zirconate ceramics. J. Solid State Chem..

[14] Fridkin VM (1984). Review of recent work on the bulk photovoltaic effect in ferro and piezoelectrics. Ferroelectrics.

[15] Giocondi JL, Rohrer GS (2001). Spatially selective photochemical reduction of silver on the surface of ferroelectric barium titanate. Chem. Mater..

[16] Giocondi JL, Rohrer GS (2001). Spatial separation of photochemical oxidation and reduction reactions on the surface of ferroelectric BaTiO_3_. J. Phys. Chem. B.

[17] Bhardwaj A, Burbure NV, Gamalski A, Rohrer GS (2010). Composition dependence of the photochemical reduction of Ag by Ba_1-x_Sr_x_TiO_3_. Chem. Mater..

[18] Wu W, Wen X, Wang ZL (2013). Taxel-addressable matrix of vertical-nanowire piezotronic transistors for active and adaptive tactile imaging. Science.

[19] Zhang Y, Xie MY, Adamaki V, Khanbareh H, Bowen CR (2017). Control of electro-chemical processes using energy harvesting materials and devices. Chem. Soc. Rev..

[20] Sebald G, Guyomar D, Agbossou A (2009). On thermoelectric and pyroelectric energy harvesting. Smart Mater. Struct..

[21] Fei ZY, Zhao WJ, Palomaki TA, Sun BS, Miller MK (2018). Ferroelectric switching of a two-dimensional metal. Nature.

[22] Li W, Ji LJ (2018). Perovskite ferroelectrics go metal free. Science.

[23] Ahn CH, Rabe KM, Triscone JM (2004). Ferroelectricity at the nanoscale: local polarization in oxide thin films and heterostructures. Science.

[24] You YM, Liao WQ, Zhao DW, Ye HY, Zhang Y (2017). An organic-inorganic perovskite ferroelectric with large piezoelectric response. Science.

[25] Wang ZL, Song JH (2006). Piezoelectric nanogenerators based on zinc oxide nanowire arrays. Science.

[26] Masmanidis SC, Karabalin RB, De Vlaminck I, Borghs G, Freeman MR, Roukes ML (2007). Multifunctional nanomechanical systems via tunably coupled piezoelectric actuation. Science.

[27] Starr MB, Wang XD (2013). Fundamental analysis of piezocatalysis process on the surfaces of strained piezoelectric materials. Sci. Rep..

[28] Fujishima A, Honda K (1972). Electrochemical photolysis of water at a semiconductor electrode. Nature.

[29] Das D, Veziroglu TN (2001). Hydrogen production by biological processes: a survey of literature. Int. J. Hydrog. Energy.

[30] Grigoriev SA, Porembsky VI, Fateev VN (2006). Pure hydrogen production by PEM electrolysis for hydrogen energy. Int. J. Hydrog. Energy.

[31] Mostafaeipour A, Khayyami M, Sedaghat A, Mohammadi K, Shamshirband S, Sehati MA, Gorakifard E (2016). Evaluating the wind energy potential for hydrogen production: a case study. Int. J. Hydrog. Energy.

[32] Hong KS, Xu HF, Konishi H, Li XC (2010). Direct water splitting through vibrating piezoelectric microfibers in water. J. Phys. Chem. Lett..

[33] Jacobson MZ, Masters GM (2001). Energy: exploiting wind versus coal. Science.

[34] Metz WD (1977). Wind energy: large and small systems competing. Science.

[35] Qolipour M, Mostafaeipour A, Tousi OM (2017). Techno-economic feasibility of a photovoltaic-wind power plant construction for electric and hydrogen production: a case study. Renew. Sustain. Energy Rev..

[36] Li HD, Sang YH, Chang SJ, Huang X, Zhang Y (2015). Enhanced ferroelectric-nanocrystal-based hybrid photocatalysis by ultrasonic-wave-generated piezophototronic effect. Nano Lett..

[37] Qian W, Zhao K, Zhang D, Bowen CR, Wang Y, Yang Y (2019). Piezoelectric material-polymer composite porous foam for efficient dye degradation via the piezo-catalytic effect. ACS Appl. Mater. Interfaces..

[38] Zhang YL, Schultz AM, Salvador PA, Rohrer GS (2011). Spatially selective visible light photocatalytic activity of TiO_2_/BiFeO_3_ heterostructures. J. Mater. Chem..

[39] Bermudez V, Caccavale F, Sada C, Segato F, Dieguez E (1998). Etching effect on periodic domain structures of lithium niobate crystals. J. Cryst. Growth.

[40] Kubel F, Schmid H (1993). Growth, twinning and etch figures of ferroelectric ferroelastic dendritic BiFeO3 single domain crystals. J. Cryst. Growth.

[41] Holstein WL (1997). Etching study of ferroelectric microdomains in LiNbO_3_ and MgO:LiNbO_3_. J. Cryst. Growth.

[42] Gorobets OY, Gorobets SV, Dvoynenko OK (2012). Correlation between the sizing features of domain structure and the etching figures of a steel cylinder. Metallofiz Nov. Tekh..

[43] Sones CL, Mailis S, Brocklesby WS, Eason RW, Owen JR (2002). Differential etch rates in z-cut LiNbO3 for variable HF/HNO3 concentrations. J. Mater. Chem..

[44] Xue XY, Wang SH, Guo WX, Zhang Y, Wang ZL (2012). Hybridizing energy conversion and storage in a mechanical-to-electrochemical process for self-charging power cell. Nano Lett..

[45] Ramadoss A, Saravanakumar B, Lee SW, Kim YS, Kim SJ, Wang ZL (2015). Piezoelectric-driven self-charging supercapacitor power cell. ACS Nano.

[46] Song RB, Jin HY, Li X, Fei LF, Zhao YD (2015). A rectification-free piezo-supercapacitor with a polyvinylidene fluoride separator and functionalized carbon cloth electrodes. J. Mater. Chem. A.

[47] Wang F, Jiang CM, Tang CL, Bi S, Wang QH, Du DF, Song JH (2016). High output nano-energy cell with piezoelectric nanogenerator and porous supercapacitor dual functions—a technique to provide sustaining power by harvesting intermittent mechanical energy from surroundings. Nano Energy.

[48] Parida K, Bhavanasi V, Kumar V, Wang JX, Lee PS (2017). Fast charging self-powered electric double layer capacitor. J. Power Sources.

[49] Lee BS, Yoon J, Jung C, Kim DY, Jeon SY (2016). Silicon/carbon nanotube/BaTiO3 nanocomposite anode: evidence for enhanced lithium-ion mobility induced by the local piezoelectric potential. ACS Nano.

[50] Kim S, Choi SJ, Zhao KJ, Yang H, Gobbi G, Zhang SL, Li J (2016). Electrochemically driven mechanical energy harvesting. Nat. Commun..

[51] Lang SB, Tofail SAM, Kholkin AL, Wojtas M, Gregor M (2013). Ferroelectric polarization in nanocrystalline hydroxyapatite thin films on silicon. Sci. Rep..

[52] Baxter FR, Bowen CR, Turner IG, Dent ACE (2010). Electrically active bioceramics: a review of interfacial responses. Ann. Biomed. Eng..

[53] Touach N, Ortiz-Martinez VM, Salar-Garcia MJ, Benzaouak A, Hernandez-Fernandez F (2017). On the use of ferroelectric material LiNbO_3_ as novel photocatalyst in wastewater-fed microbial fuel cells. Particuology.

[54] Jacques E, Kjell MH, Zenkert D, Lindbergh G (2013). Piezo-electrochemical effect in lithium-intercalated carbon fibres. Electrochem. Commun..

[55] Jacques E, Lindbergh G, Zenkert D, Leijonmarck S, Kjell MH (2015). Piezo-electrochemical energy harvesting with lithium-intercalating carbon fibers. ACS Appl. Mater. Interfaces..

[56] Ikeda S, Takata T, Kondo T, Hitoki G, Hara M (1998). Mechano-catalytic overall water splitting. Chem. Commun..

[57] Asadian E, Ghalkhani M, Shahrokhian S (2019). Electrochemical sensing based on carbon nanoparticles: a review. Sens. Actuator B.

[58] Rossner L, Armbruster M (2019). Electrochemical energy conversion on intermetallic compounds: a review. ACS Catal..

[59] Vogiazi V, de la Cruz A, Mishra S, Shanov V, Heineman WR, Dionysiou DD (2019). A comprehensive review: development of electrochemical biosensors for detection of cyanotoxins in freshwater. ACS Sens..

[60] Kim J, Kumar R, Bandodkar AJ, Wang J (2017). Advanced materials for printed wearable electrochemical devices: a review. Adv. Electron. Mater..

[61] Fresta E, Costa RD (2017). Beyond traditional light-emitting electrochemical cells—a review of new device designs and emitters. J. Mater. Chem. C.

[62] Tian XC, Jin J, Yuan SQ, Chua CK, Tor SB, Zhou K (2017). Emerging 3D-printed electrochemical energy storage devices: a critical review. Adv. Energy Mater..

[63] Nayak PK, Erickson EM, Schipper F, Penki TR, Munichandraiah N (2018). Review on challenges and recent advances in the electrochemical performance of high capacity Li- and Mn-rich cathode materials for li-ion batteries. Adv. Energy Mater..

[64] Saratale RG, Saratale GD, Pugazhendhi A, Zhen GY, Kumar G, Kadier A, Sivagurunathan P (2017). Microbiome involved in microbial electrochemical systems (MESs): a review. Chemosphere.

[65] Starr MB, Shi J, Wang XD (2012). Piezopotential-driven redox reactions at the surface of piezoelectric materials. Angew. Chem. Int. Ed..

[66] Zhang J, Wu Z, Jia YM, Kan JW, Cheng GM (2013). Piezoelectric bimorph cantilever for vibration-producing-hydrogen. Sensors.

[67] Lin H, Wu Z, Jia YM, Li WJ, Zheng RK, Luo HS (2014). Piezoelectrically induced mechano-catalytic effect for degradation of dye wastewater through vibrating Pb(Zr_0.52_Ti_0.48_)O_3_ fibers. Appl. Phys. Lett..

[68] Lo MK, Lee SY, Chang KS (2015). Study of ZnSnO_3_-nanowire piezophotocatalyst using two-step hydrothermal synthesis. J. Phys. Chem. C.

[69] Tan CF, Ong WL, Ho GW (2015). Self-biased hybrid piezoelectric-photoelectrochemical cell with photocatalytic functionalities. ACS Nano.

[70] You HL, Jia YM, Wu Z, Xu XL, Qian WQ, Xia YT, Ismail M (2017). Strong piezo-electrochemical effect of multiferroic BiFeO_3_ square micro-sheets for mechanocatalysis. Electrochem. Commun..

[71] You HL, Wu Z, Zhang LH, Ying YR, Liu Y (2019). Harvesting the vibration energy of BiFeO_3_ nanosheets for hydrogen evolution. Angew. Chem. Int. Ed..

[72] Wu JM, Chang WE, Chang YT, Chang CK (2016). Piezo-catalytic effect on the enhancement of the ultra-high degradation activity in the dark by single- and few-layers MoS_2_ nanoflowers. Adv. Mater..

[73] Lin JH, Tsao YH, Wu MH, Chou TM, Lin ZH, Wu JM (2017). Single- and few-layers MoS_2_ nanocomposite as piezo-catalyst in dark and self-powered active sensor. Nano Energy.

[74] Burbure NV, Salvador PA, Rohrer GS (2010). Photochemical reactivity of titania films on BaTiO_3_ substrates: origin of spatial selectivity. Chem. Mater..

[75] Lan SY, Feng JX, Xiong Y, Tian SH, Liu SW, Kong LJ (2017). Performance and mechanism of piezo-catalytic degradation of 4-chlorophenol: finding of effective piezo-dechlorination. Environ. Sci. Technol..

[76] Lv W, Kong LJ, Lan SY, Feng JX, Xiong Y, Tian SH (2017). Enhancement effect in the piezoelectric degradation of organic pollutants by piezo-Fenton process. J. Chem. Technol. Biotechnol..

[77] Tiwari D, Dunn S, Zhang Q (2009). Impact of Zr/Ti ratio in the PZT on the photoreduction of silver nanoparticles. Mater. Res. Bull..

[78] Mushtaq F, Chen X, Hoop M, Torlakcik H, Pellicer E (2018). Piezoelectrically enhanced photocatalysis with BiFeO_3_ nanostructures for efficient water remediation. Science.

[79] Sun C, Fu YM, Wang Q, Xing LL, Liu BD, Xue XY (2016). Ultrafast piezo-photocatalytic degradation of organic pollutions by Ag_2_O/tetrapod-ZnO nanostructures under ultrasonic/UV exposure. RSC Adv..

[80] Hong DY, Zang WL, Guo X, Fu YM, He HX (2016). High piezo-photocatalytic efficiency of CuS/ZnO nanowires using both solar and mechanical energy for degrading organic dye. ACS Appl. Mater. Interfaces..

[81] Wu MH, Lee JT, Chung YJ, Srinivaas M, Wu JM (2017). Ultrahigh efficient degradation activity of single- and few-layered MoSe_2_ nanoflowers in dark by piezo-catalyst effect. Nano Energy.

[82] Masimukku S, Hu YC, Lin ZH, Chan SW, Chou TM, Wu JM (2018). High efficient degradation of dye molecules by PDMS embedded abundant single-layer tungsten disulfide and their antibacterial performance. Nano Energy.

[83] Xing LL, Nie YX, Xue XY, Zhang Y (2014). PVDF mesoporous nanostructures as the piezo-separator for a self-charging power cell. Nano Energy.

[84] Kim YS, Xie YN, Wen XN, Wang SH, Kim SJ, Song HK, Wang ZL (2015). Highly porous piezoelectric PVDF membrane as effective lithium ion transfer channels for enhanced self-charging power cell. Nano Energy.

[85] Noris-Suarez K, Lira-Olivares J, Ferreira AM, Graterol A, Feijoo JL, Lee SW (2007). Electrochemical influence of collagen piezoelectric effect in bone healing. Mater. Sci. Forum.

[86] Giocondi JL, Rohrer GS (2008). The influence of the dipolar field effect on the photochemical reactivity of Sr_2_Nb_2_O_7_ and BaTiO_3_ microcrystals. Top. Catal..

[87] Hong KS, Xu HF, Konishi H, Li XC (2012). Piezoelectrochemical effect: a new mechanism for azo dye decolorization in aqueous solution through vibrating piezoelectric microfibers. J. Phys. Chem. C.

[88] Xue XY, Zang WL, Deng P, Wang Q, Xing LL, Zhang Y, Wang ZL (2015). Piezo-potential enhanced photocatalytic degradation of organic dye using ZnO nanowires. Nano Energy.

[89] Wang YT, Chang KS (2016). Piezopotential-induced schottky behavior of Zn_1-x_SnO_3_ nanowire arrays and piezophotocatalytic applications. J. Am. Ceram. Soc..

[90] Zhang HL, Zhang SJ, Yao G, Huang ZL, Xie YH (2015). Simultaneously harvesting thermal and mechanical energies based on flexible hybrid nanogenerator for self-powered cathodic protection. ACS Appl. Mater. Interfaces..

[91] Bai XF, Wei J, Tian BB, Liu Y, Reiss T (2016). Size effect on optical and photocatalytic properties in BiFeO_3_ nanoparticles. J. Phys. Chem. C.

[92] Park TJ, Papaefthymiou GC, Viescas AJ, Moodenbaugh AR, Wong SS (2007). Size-dependent magnetic properties of single-crystalline multiferroic BiFeO_3_ nanoparticles. Nano Lett..

[93] Li S, Lin YH, Zhang BP, Wang Y, Nan CW (2010). Controlled fabrication of BiFeO_3_ uniform microcrystals and their magnetic and photocatalytic behaviors. J. Phys. Chem. C.

[94] Espinosa HD, Bernal RA, Minary-Jolandan M (2012). A review of mechanical and electromechanical properties of piezoelectric nanowires. Adv. Mater..

[95] Singh SK, Ishiwara H, Maruyama K (2006). Room temperature ferroelectric properties of Mn-substituted BiFeO_3_ thin films deposited on Pt electrodes using chemical solution deposition. Appl. Phys. Lett..

[96] Park JM, Nakashima S, Sohgawa M, Kanashima T, Okuyama M (2012). Ferroelectric and piezoelectric properties of polycrystalline BiFeO_3_ thin films prepared by pulsed laser deposition under magnetic field. Jpn. J. Appl. Phys..

[97] Mantini G, Gao YF, D’Amico A, Falconi C, Wang ZL (2009). Equilibrium piezoelectric potential distribution in a deformed ZnO nanowire. Nano Res..

[98] Choi MY, Choi D, Jin MJ, Kim I, Kim SH, Choi JY (2009). Mechanically powered transparent flexible charge-generating nanodevices with piezoelectric ZnO nanorods. Adv. Mater..

[99] Gao ZY, Zhou J, Gu YD, Fei P, Hao Y, Bao G, Wang ZL (2009). Effects of piezoelectric potential on the transport characteristics of metal-ZnO nanowire-metal field effect transistor. J. Appl. Phys..

[100] Feng YW, Ling LL, Wang YX, Xu ZM, Cao FL, Li HX, Bian ZF (2017). Engineering spherical lead zirconate titanate to explore the essence of piezo-catalysis. Nano Energy.

[101] Wu J, Qin N, Bao DH (2018). Effective enhancement of piezocatalytic activity of BaTiO3 nanowires under ultrasonic vibration. Nano Energy.

[102] M.C. Junger, D. Feit, *Sound, Structures, and Their Interaction*, 2nd ed. (MIT Press, Cambridge, 1986). http://hdl.handle.net/1721.1/1740

[103] Gulin OE, Klyatskin VI (1993). Generation of a low-frequency acoustical noise in the layered ocean by the surface sources. Nat. Phys. Sources Underw. Sound.

[104] Scrimger JA, Evans DJ, Yee W (1989). Underwater noise due to rain—open ocean measurements. J. Acoust. Soc. Am..

[105] Adewuyi YG (2001). Sonochemistry: environmental science and engineering applications. Ind. Eng. Chem. Res..

[106] Ince NH, Tezcanli G, Belen RK, Apikyan IG (2001). Ultrasound as a catalyzer of aqueous reaction systems: the state of the art and environmental applications. Appl. Catal. B.

[107] Tezcanli-Guyer G, Ince NH (2004). Individual and combined effects of ultrasound, ozone and UV irradiation: a case study with textile dyes. Ultrasonics.

[108] Li YF, Liu ZP (2011). Particle size, shape and activity for photocatalysis on titania anatase nanoparticles in aqueous surroundings. J. Am. Chem. Soc..

[109] You HL, Wu Z, Jia YM, Xu XL, Xia YT, Han ZC, Wang Y (2017). High-efficiency and mechano-/photo-bi-catalysis of piezoelectric-ZnO@ photoelectric-TiO_2_ core-shell nanofibers for dye decomposition. Chemosphere.

[110] Rai SC, Wang K, Chen JJ, Marmon JK, Bhatt M (2015). Enhanced broad band photodetection through piezo-phototronic effect in CdSe/ZnTe core/shell nanowire array. Adv. Electron. Mater..

[111] Han C, Yang MQ, Zhang N, Xu YJ (2014). Enhancing the visible light photocatalytic performance of ternary CdS-(graphene-Pd) nanocomposites via a facile interfacial mediator and co-catalyst strategy. J. Mater. Chem. A.

[112] Brandt RE, Young M, Park HH, Dameron A, Chua D (2014). Band offsets of n-type electron-selective contacts on cuprous oxide (Cu_2_O) for photovoltaics. Appl. Phys. Lett..

[113] Meng LJ, Ma AF, Ying PL, Feng ZC, Li C (2011). Sputtered highly ordered TiO_2_ nanorod arrays and their applications as the electrode in dye-sensitized solar cells. J. Nanosci. Nanotechnol..

[114] Banerjee S, Pillai SC, Falaras P, O’Shea KE, Byrne JA, Dionysiou DD (2014). New insights into the mechanism of visible light photocatalysis. J. Phys. Chem. Lett..

[115] Chen CC, Lei PX, Ji HW, Ma WH, Zhao JC, Hidaka H, Serpone N (2004). Photocatalysis by titanium dioxide and polyoxometalate/TiO2 cocatalysts. Intermediates and mechanistic study. Environ. Sci. Technol..

[116] Shi J, Starr MB, Xiang H, Hara Y, Anderson MA, Seo JH, Ma ZQ, Wang XD (2011). Interface engineering by piezoelectric potential in ZnO-based photoelectrochemical anode. Nano Lett..

[117] Lu LW, Lv ML, Liu G, Xu XX (2017). Photocatalytic hydrogen production over solid solutions between BiFeO_3_ and SrTiO_3_. Appl. Surf. Sci..

[118] Daneshvar N, Salari D, Khataee AR (2004). Photocatalytic degradation of azo dye acid red 14 in water on ZnO as an alternative catalyst to TiO_2_. J. Photochem. Photobiol., A.

[119] Qian WQ, Wu Z, Jia YM, Hong YT, Xu XL (2017). Thermo-electrochemical coupling for room temperature thermocatalysis in pyroelectric ZnO nanorods. Electrochem. Commun..

[120] Konstantinou IK, Albanis TA (2004). TiO_2_-assisted photocatalytic degradation of azo dyes in aqueous solution: kinetic and mechanistic investigations: a review. Appl. Catal. B.

[121] Galindo C, Jacques P, Kalt A (2001). Photooxidation of the phenylazonaphthol AO_20_ on TIO_2_: kinetic and mechanistic investigations. Chemosphere.

[122] Bandara J, Mielczarski JA, Kiwi J (1999). Photosensitized degradation of azo dyes on Fe, Ti, and Al oxides. Mechanism of charge transfer during the degradation. Langmuir.

[123] Stylidi M, Kondarides DI, Verykios XE (2003). Pathways of solar light-induced photocatalytic degradation of azo dyes in aqueous TiO_2_ suspensions. Appl. Catal. B.

[124] Jaffe B, Roth RS, Marzullo S (1954). Piezoelectric properties of lead zirconate-lead titanate solid-solution ceramics. J. Appl. Phys..

[125] Damjanovic D (2009). Comments on origins of enhanced piezoelectric properties in ferroelectrics. IEEE Trans. Ultrason. Ferroelectr..

[126] Liao WQ, Zhao DW, Tang YY, Zhang Y, Li PF (2019). A molecular perovskite solid solution with piezoelectricity stronger than lead zirconate titanate. Science.

[127] Borman TM, Ko SW, Mardilovich P, Trolier-McKinstry SE (2017). Development of crystallographic texture in chemical solution deposited lead zirconate titanate seed layers. J. Am. Ceram. Soc..

[128] Nguyen MD, Houwman EP, Dekkers M, Rijnders G (2017). Strongly enhanced piezoelectric response in lead zirconate titanate films with vertically aligned columnar grains. ACS Appl. Mater. Interfaces..

[129] Schatz A, Pantel D, Hanemann T (2017). Pulsed laser deposition of piezoelectric lead zirconate titanate thin films maintaining a post-CMOS compatible thermal budget. J. Appl. Phys..

[130] Shrout TR, Zhang SJ (2007). Lead-free piezoelectric ceramics: Alternatives for PZT?. J. Electroceram..

[131] Brajesh K, Tanwar K, Abebe M, Ranjan R (2015). Relaxor ferroelectricity and electric-field-driven structural transformation in the giant lead-free piezoelectric (Ba, Ca)(Ti, Zr)O3. Phys. Rev. B.

[132] Cheng RF, Xu ZJ, Chu RQ, Hao JG, Du J, Li GR (2015). Structure and electrical properties of Bi_1/2_Na_1/2_TiO_3_-based lead-free piezoelectric ceramics. RSC Adv..

[133] Kim KN, Chun J, Chae SA, Ahn CW, Kim IW (2015). Silk fibroin-based biodegradable piezoelectric composite nanogenerators using lead-free ferroelectric nanoparticles. Nano Energy.

[134] Wei HG, Wang H, Xia YJ, Cui DP, Shi YP (2018). An overview of lead-free piezoelectric materials and devices. J. Mater. Chem. C.

[135] Chen J, Oh SK, Zou HY, Shervin S, Wang WJ (2018). High-output lead-free flexible piezoelectric generator using single-crystalline GaN thin film. ACS Appl. Mater. Interfaces..

[136] Akbarzadeh A, Brajesh K, Nahas Y, Kumar N, Prokhorenko S (2018). Quantum-fluctuation-stabilized orthorhombic ferroelectric ground state in lead-free piezoelectric (Ba, Ca)(Zr, Ti)O_3_. Phys. Rev. B.

[137] Wang YM, Wu HJ, Qin X, Yao K, Pennycook SJ, Tay FEH (2019). Outstanding piezoelectric performance in lead-free 0.95(K, Na)(Sb, Nb)O-3-0.05(Bi, Na, K)ZrO3 thick films with oriented nanophase coexistence. Adv. Electron. Mater..

[138] Rodel J, Jo W, Seifert KTP, Anton EM, Granzow T, Damjanovic D (2009). Perspective on the development of lead-free piezoceramics. J. Am. Ceram. Soc..

[139] Kushvaha DK, Rout SK, Tiwari B (2019). Structural, piezoelectric and highdensity energy storage properties of lead-free BNKT-BCZT solid solution. J. Alloys Compd..

[140] Moure C, Pena O (2015). Recent advances in perovskites: processing and properties. Prog. Solid State Chem..

[141] Mathews S, Ramesh R, Venkatesan T, Benedetto J (1997). Ferroelectric field effect transistor based on epitaxial perovskite heterostructures. Science.

[142] Choi KJ, Biegalski M, Li YL, Sharan A, Schubert J, Uecker R (2004). Enhancement of ferroelectricity in strained BaTiO_3_ thin films. Science.

[143] Zheng H, Wang J, Lofland SE, Ma Z, Mohaddes-Ardabili L (2004). Multiferroic BaTiO_3_-CoFe_2_O_4_ nanostructures. Science.

[144] Jonker GH, Vansanten JH (1949). Properties of barium titanate in connection with its crystal structure. Science.

[145] Kay HF, Rhodes RG (1947). Barium titanate crystals. Nature.

[146] Wang J, Neaton JB, Zheng H, Nagarajan V, Ogale SB (2003). Epitaxial BiFeO_3_ multiferroic thin film heterostructures. Science.

[147] Eerenstein W, Morrison FD, Dho J, Blamire MG, Scott JF, Mathur ND (2005). Comment on “Epitaxial BiFeO_3_ multiferroic thin film heterostructures”. Science.

[148] Wang J, Scholl A, Zheng H, Ogale SB, Viehland D (2005). Response to comment on “Epitaxial BiFeO_3_ multiferroic thin film heterostructures”. Science.

[149] Zeches RJ, Rossell MD, Zhang JX, Hatt AJ, He Q (2009). A strain-driven morphotropic phase boundary in BiFeO_3_. Science.

[150] Sun JH, Dong SY, Wang YK, Sun SP (2009). Preparation and photocatalytic property of a novel dumbbell-shaped ZnO microcrystal photocatalyst. J. Hazard. Mater..

[151] Xia YT, Jia YM, Qian WQ, Xu XL, Wu Z (2017). Pyroelectrically induced pyro-electro-chemical catalytic activity of BaTiO_3_ nanofibers under room-temperature cold-hot cycle excitations. Metals.

[152] Yuan SG, Yang ZB, Xie C, Yan F, Dai JY (2016). Ferroelectric-driven performance enhancement of graphene field-effect transistors based on vertical tunneling heterostructures. Adv. Mater..

[153] Wong MC, Chen L, Tsang MK, Zhang Y, Hao JH (2015). Magnetic-induced luminescence from flexible composite laminates by coupling magnetic field to piezophotonic effect. Adv. Mater..

[154] Ma JP, Ren J, Jia YM, Wu Z, Chen L (2019). High efficiency bi-harvesting light/vibration energy using piezoelectric zinc oxide nanorods for dye decomposition. Nano Energy.

[155] Khan A, Abbasi MA, Hussain M, Ibupoto ZH, Wissting J, Nur O, Willander M (2012). Piezoelectric nanogenerator based on zinc oxide nanorods grown on textile cotton fabric. Appl. Phys. Lett..

[156] Mahmud A, Khan AA, Voss P, Das T, Abdel-Rahman E, Ban D (2018). A high performance and consolidated piezoelectric energy harvester based on 1D/2D hybrid zinc oxide nanostructures. Adv. Mater. Interfaces.

[157] Wang ZW, Zhang ZM, Liu W, Wang ZL (2017). Investigating fold structures of 2D materials by quantitative transmission electron microscopy. Micron.

[158] Duerloo KAN, Ong MT, Reed EJ (2012). Intrinsic piezoelectricity in two-dimensional materials. J. Phys. Chem. Lett..

[159] Huang KJ, Wang L, Liu YJ, Wang HB, Liu YM, Wang LL (2013). Synthesis of polyaniline/2-dimensional graphene analog MoS_2_ composites for high-performance supercapacitor. Electrochim. Acta.

[160] Mann J, Ma Q, Odenthal PM, Isarraraz M, Le D (2014). 2-Dimensional transition metal dichalcogenides with tunable direct band gaps: MoS2_(1-x)_Se_2x_ monolayers. Adv. Mater..

[161] Rao CNR, Maitra U, Waghmare UV (2014). Extraordinary attributes of 2-dimensional MoS_2_ nanosheets. Chem. Phys. Lett..

[162] Shi SL, Sun ZX, Hu YH (2018). Synthesis, stabilization and applications of 2-dimensional 1T metallic MoS_2_. J. Mater. Chem. A.

[163] Nowakowski K, van Bremen R, Zandvliet HJW, Bampoulis P (2019). Control of the metal/WS_2_ contact properties using 2-dimensional buffer layers. Nanoscale.

[164] Yu X, Pei CG, Chen WS, Feng LG (2018). 2 dimensional WS_2_ tailored nitrogen-doped carbon nanofiber as a highly pseudocapacitive anode material for lithium-ion battery. Electrochim. Acta.

[165] Xu SJ, Lei ZY, Wu PY (2015). Facile preparation of 3D MoS_2_/MoSe_2_ nanosheet-graphene networks as efficient electrocatalysts for the hydrogen evolution reaction. J. Mater. Chem. A.

[166] Chen XS, Liu GB, Zheng W, Feng W, Cao WW, Hu WP, Hu PA (2016). Vertical 2D MoO_2_/MoSe_2_ core-shell nanosheet arrays as high-performance electrocatalysts for hydrogen evolution reaction. Adv. Funct. Mater..

[167] Geng XS, Yu YQ, Zhou XL, Wang CD, Xu KW (2016). Design and construction of ultra-thin MoSe_2_ nanosheet-based heterojunction for high-speed and low-noise photodetection. Nano Res..

[168] Peng H, Wei CD, Wang K, Meng TY, Ma GF, Lei ZQ, Gong X (2017). Ni_0.85_Se@MoSe_2_ nanosheet arrays as the electrode for high-performance supercapacitors. ACS Appl. Mater. Interfaces..

[169] He LZ, Nie TQ, Xia XJ, Liu T, Huang YY, Wang XJ, Chen TF (2019). Designing bioinspired 2D MoSe_2_ nanosheet for efficient photothermal-triggered cancer immunotherapy with reprogramming tumor-associated macrophages. Adv. Funct. Mater..

[170] Cauda V, Stassi S, Bejtka K, Canayese G (2013). Nanoconfinement: an effective way to enhance PVDF piezoelectric properties. ACS Appl. Mater. Interfaces..

[171] Pi ZY, Zhang JW, Wen CY, Zhang ZB, Wu DP (2014). Flexible piezoelectric nanogenerator made of poly(vinylidenefluoride-co-trifluoroethylene) (PVDF-TrFE) thin film. Nano Energy.

[172] Alluri NR, Saravanakumar B, Kim SJ (2015). Flexible, hybrid piezoelectric film (BaTi_(1-x)_Zr_x_O_3_)/PVDF nanogenerator as a self-powered fluid velocity sensor. ACS Appl. Mater. Interfaces..

[173] Karan SK, Mandal D, Khatua BB (2015). Self-powered flexible Fe-doped RGO/PVDF nanocomposite: an excellent material for a piezoelectric energy harvester. Nanoscale.

[174] Tian G, Deng WL, Gao YY, Xiong D, Yan C (2019). Rich lamellar crystal baklava-structured PZT/PVDF piezoelectric sensor toward individual table tennis training. Nano Energy.

[175] Li QQ, Ke WY, Chang TX, Hu ZJ (2019). A molecular ferroelectrics induced electroactive β-phase in solution processed PVDF films for flexible piezoelectric sensors. J. Mater. Chem. C.

[176] Knott L, Bailey AJ (1998). Collagen cross-links in mineralizing tissues: a review of their chemistry, function, and clinical relevance. Bone.

[177] Silva CC, Lima CGA, Pinheiro AG, Goes JC, Figueiro SD, Sombra ASB (2001). On the piezoelectricity of collagen-chitosan film. Phys. Chem. Chem. Phys..

[178] Ghosh SK, Mandal D (2017). Sustainable energy generation from piezoelectric biomaterial for noninvasive physiological signal monitoring. ACS Sustain. Chem. Eng.

[179] Jones PM, Gallardo DE, Dunn S (2008). Photochemical investigation of a polarizable semiconductor, lead-zirconate-titanate. Chem. Mater..

[180] Xue XY, Deng P, Yuan S, Nie YX, He B, Xing LL, Zhang Y (2013). CuO/PVDF nanocomposite anode for a piezo-driven self-charging lithium battery. Energy Environ. Sci..

[181] Zhang Y, Zhang YJ, Xue XY, Cui CX, He B (2014). PVDF-PZT nanocomposite film based self-charging power cell. Nanotechnology.

[182] Wang ZY, Hu J, Yu MF (2006). One-dimensional ferroelectric monodomain formation in single crystalline BaTiO_3_ nanowire. Appl. Phys. Lett..

[183] Yu D, Zhao ML, Wang CL, Wang LH, Su WB (2016). Enhanced piezoelectricity in plastically deformed nearly amorphous Bi_12_TiO_20_-BaTiO_3_ nanocomposites. Appl. Phys. Lett..

[184] Xu XL, Jia YM, Xiao LB, Wu Z (2018). Strong vibration-catalysis of ZnO nanorods for dye wastewater decolorization via piezo-electro-chemical coupling. Chemosphere.

[185] Guralnick SM (1979). Contexts of faraday electrochemical laws. Isis.

[186] Soroushian P, Nassar RUD, Balachandra AM (2013). Piezo-driven self-healing by electrochemical phenomena. J. Intell. Mater. Syst. Struct..

[187] Ribeiro C, Sencadas V, Correia DM, Lanceros-Mendez S (2015). Piezoelectric polymers as biomaterials for tissue engineering applications. Colloid Surface B.

[188] Shamos MH, Lavine LS (1967). Piezoelectricity as a fundamental property of biological tissues. Nature.

[189] Athenstaedt H (1971). Pyroelectric and piezoelectric behaviour of human dental hard tissues. Arch. Oral Biol..

[190] Hofkens J, Roeffaers MBJ (2016). Electrochemistry: photocatalysts in close-up. Nature.

[191] Richards JW (1904). The continuous advance of electrochemistry. Science.

[192] Fridkin VM (1979). Photoferroelectrics.

[193] Bard AJ, Bocarsly AB, Fan FRF, Walton EG, Wrighton MS (1980). The concept of Fermi level pinning at semiconductor-liquid junctions—consequences for energy-conversion efficiency and selection of useful solution redox couples in solar devices. J. Am. Chem. Soc..

[194] Dunn S, Tiwari D, Jones PM, Gallardo DE (2007). Insights into the relationship between inherent materials properties of PZT and photochemistry for the development of nanostructured. J. Mater. Chem..

[195] Smith WA, Sharp ID, Strandwitz NC, Bisquert J (2015). Interfacial band-edge energetics for solar fuels production. Energy Environ. Sci..

[196] Salvador P (2001). Semiconductors’ photoelectrochemistry: a kinetic and thermodynamic analysis in the light of equilibrium and nonequilibrium models. J. Phys. Chem. B.

[197] Wrighton MS, Ellis AB, Wolczanski PT, Morse DL, Abrahamson HB, Ginley DS (1976). Strontium titanate photoelectrodes. Efficient photoassisted electrolysis of water at zero applied potential. J. Am. Chem. Soc..

[198] Green L (1967). Energy needs versus environmental pollution: a reconciliation?. Science.

[199] Berg D, Hickman RG, Kovats A (1970). Energy without pollution. Science.

[200] Turner JA (2004). Sustainable hydrogen production. Science.

[201] Park S, Chang WJ, Lee CW, Park S, Ahn HY, Nam KT (2017). Photocatalytic hydrogen generation from hydriodic acid using methylammonium lead iodide in dynamic equilibrium with aqueous solution. Nat. Energy.

[202] Zhang P, Guo YJ, Chen JB, Zhao YR, Chang J, Junge H, Beller M, Li Y (2018). Streamlined hydrogen production from biomass. Nat. Catal..

[203] Sartbaeva A, Kuznetsov VL, Wells SA, Edwards PP (2008). Hydrogen nexus in a sustainable energy future. Energy Environ. Sci..

[204] Lin L, Zhou W, Gao R, Yao S, Zhang X (2017). Low-temperature hydrogen production from water and methanol using Pt/alpha-MoC catalysts. Nature.

[205] Hu Q, Kim DY, Yang W, Yang L, Meng Y, Zhang L, Mao HK (2016). FeO_2_ and FeOOH under deep lower-mantle conditions and Earth’s oxygen-hydrogen cycles. Nature.

[206] Lachheb H, Puzenat E, Houas A, Ksibi M, Elaloui E, Guillard C, Herrmann JM (2002). Photocatalytic degradation of various types of dyes (Alizarin S, Crocein Orange G, Methyl Red, Congo Red, Methylene Blue) in water by UV-irradiated titania. Appl. Catal. B.

[207] Petrolekas PD, Maggenakis G (2007). Kinetic studies of the liquid-phase adsorption of a reactive dye onto activated lignite. Ind. Eng. Chem. Res..

[208] Li Q, Yue QY, Su Y, Gao BY, Li J (2009). Two-step kinetic study on the adsorption and desorption of reactive dyes at cationic polymer/bentonite. J. Hazard. Mater..

[209] Li J, Cai J, Zhong L, Wang H, Cheng H, Ma Q (2018). Adsorption of reactive dyes onto chitosan/montmorillonite intercalated composite: multi-response optimization, kinetic, isotherm and thermodynamic study. Water Sci. Technol..

[210] Khan HM, Anwar M, Ahmad G (1995). Effect of temperature and light on the response of an aqueous coumarin dosimeter. J. Radioanal. Nucl. Chem. Lett..

[211] Gao F, Chen XY, Yin KB, Dong S, Ren ZF (2007). Visible-light photocatalytic properties of weak magnetic BiFeO_3_ nanoparticles. Adv. Mater..

[212] Prevot AB, Baiocchi C, Brussino MC, Pramauro E, Savarino P (2001). Photocatalytic degradation of acid blue 80 in aqueous solutions containing TiO_2_ suspensions. Environ. Sci. Technol..

[213] Houas A, Lachheb H, Ksibi M, Elaloui E, Guillard C, Herrmann JM (2001). Photocatalytic degradation pathway of methylene blue in water. Appl. Catal. B.

[214] Patil SS, Shinde VM (1988). Biodegradation studies of aniline and nitrobenzene in aniline plant wastewater by gas chromatography. Environ. Sci. Technol..

[215] Arslan I, Balcioglu IA (1999). Degradation of commercial reactive dyestuffs by heterogenous and homogenous advanced oxidation processes: a comparative study. Dyes Pigments.

[216] Moore AT, Vira A, Fogel S (1989). Biodegradation of trans-1,2-dichloroethylene by methane-utilizing bacteria in an aquifer simulator. Environ. Sci. Technol..

[217] Borgarello E, Kiwi J, Pelizzetti E, Visca M, Gratzel M (1981). Photochemical cleavage of water by photocatalysis. Nature.

[218] Asahi R, Morikawa T, Ohwaki T, Aoki K, Taga Y (2001). Visible-light photocatalysis in nitrogen-doped titanium oxides. Science.

[219] Peng Y, Wang KK, Liu T, Xu J, Xu B (2017). Synthesis of one-dimensional Bi_2_O_3_-Bi_2_O_2.33_ heterojunctions with high interface quality for enhanced visible light photocatalysis in degradation of high-concentration phenol and MO dyes. Appl. Catal. B.

[220] Yang RS, Qin Y, Dai LM, Wang ZL (2009). Power generation with laterally packaged piezoelectric fine wires. Nat. Nanotechnol..

[221] Hu YF, Zhang Y, Xu C, Lin L, Snyder RL, Wang ZL (2011). Self-powered system with wireless data transmission. Nano Lett..

[222] Chang CE, Tran VH, Wang JB, Fuh YK, Lin LW (2010). Direct-write piezoelectric polymeric nanogenerator with high energy conversion efficiency. Nano Lett..

[223] Chan CK, Peng HL, Liu G, McIlwrath K, Zhang XF, Huggins RA, Cui Y (2008). High-performance lithium battery anodes using silicon nanowires. Nat. Nanotechnol..

[224] Idota Y, Kubota T, Matsufuji A, Maekawa Y, Miyasaka T (1997). Tin-based amorphous oxide: a high-capacity lithium-ion-storage material. Science.

[225] Tarascon JM, Armand M (2001). Issues and challenges facing rechargeable lithium batteries. Nature.

[226] Poizot P, Laruelle S, Grugeon S, Dupont L, Tarascon JM (2000). Nano-sized transition-metaloxides as negative-electrode materials for lithium-ion batteries. Nature.

[227] Bruce PG, Scrosati B, Tarascon JM (2008). Nanomaterials for rechargeable lithium batteries. Angew. Chem. Int. Ed..

[228] Armstrong G, Armstrong AR, Bruce PG, Reale P, Scrosati B (2006). TiO_2_(B) nanowires as an improved anode material for lithium-ion batteries containing LiFePO_4_ or LiNi_0.5_Mn_1.5_O_4_ cathodes and a polymer electrolyte. Adv. Mater..

[229] Mao YC, Zhao P, McConohy G, Yang H, Tong YX, Wang XD (2014). Sponge-like piezoelectric polymer films for scalable and integratable nanogenerators and self-powered electronic systems. Adv. Energy Mater..

[230] Simon P, Gogotsi Y, Dunn B (2014). Where do batteries end and supercapacitors begin?. Science.

[231] Huang P, Lethien C, Pinaud S, Brousse K, Laloo R (2016). On-chip and freestanding elastic carbon films for micro-supercapacitors. Science.

[232] Qin TF, Peng SL, Hao JX, Wen YX, Wang ZL (2017). Flexible and wearable all-solid-state supercapacitors with ultrahigh energy density based on a carbon fiber fabric electrode. Adv. Energy Mater..

[233] Cao CY, Zhou YH, Ubnoske S, Zang JF, Cao YT (2019). Highly stretchable supercapacitors via crumpled vertically aligned carbon nanotube forests. Adv. Energy Mater..

[234] Cordier P, Tournilhac F, Soulie-Ziakovic C, Leibler L (2008). Self-healing and thermoreversible rubber from supramolecular assembly. Nature.

[235] Zhang L, Bailey JB, Subramanian RH, Groisman A, Tezcan FA (2018). Hyperexpandable, self-healing macromolecular crystals with integrated polymer networks. Nature.

[236] Acome E, Mitchell SK, Morrissey TG, Emmett MB, Benjamin C (2018). Hydraulically amplified self-healing electrostatic actuators with muscle-like performance. Science.

[237] Sumerlin BS (2018). Next-generation self-healing materials. Science.

[238] Goldenberg DM, Hansen HJ (1972). Electric enhancement of bone healing. Science.

